# Sorbose metabolism promotes fitness and virulence in *Escherichia coli*

**DOI:** 10.1128/msphere.00191-26

**Published:** 2026-06-15

**Authors:** Xue Deng, Cong Shen, Lingjuan Chen, Dandan Sun, Tian Qiu, Zihan Zhao, Tong Li, Guili Zhang, Ji Wu, Juan Wang, Guo-Bao Tian, Lingqing Xu, Bin Yan, Lan-Lan Zhong

**Affiliations:** 1 Department of Immunology, Zhongshan School of Medicine, Sun Yat-sen University Shenzhen Campus, Shenzhen, China; 2 Ministry of Education, Key Laboratory of Tropical Diseases Control, Sun Yat-sen University 26469 , Guangzhou, China; 3 The Second Clinical Medical College, Guangzhou University of Chinese Medicine, State Key Laboratory of Traditional Chinese Medicine Syndrome, Guangdong Provincial Hospital of Chinese Medicine 74715, Guangzhou, China; 4 State Key Laboratory of Dampness Syndrome of Chinese Medicine/Clinical Laboratory, Guangdong Provincial Hospital of Chinese Medicine 600607, Guangzhou, China; 5 Department of Clinical Laboratory, The Affiliated Qingyuan Hospital (Qingyuan People's Hospital), Guangzhou Medical University, Qingyuan, China; 6 Guangzhou Southern College, Guangzhou, China; 7 School of Laboratory Medicine, Chengdu Medical College 74787, Chengdu, China; 8 Department of Clinical Laboratory, Shenzhen People's Hospital, Shenzhen, China; 9 Department of Neonatal Surgery, Guangzhou Women and Children’s Medical Center, Guangzhou Medical University 26468, Guangzhou, China; Hackensack Meridian Health Center for Discovery and Innovation, Nutley, New Jersey, USA

**Keywords:** virulence, fitness, *Mcr-1*-positive *Escherichia coli*, Sorbose metabolism, LPS biosynthesis, FimH

## Abstract

The plasmid-borne *mcr-1* gene poses a significant threat to global health by conferring resistance to colistin, a critical last-resort antibiotic. While its spread is well documented, the adaptations enabling its concurrent antibiotic resistance and clinical pathogenicity remain unknown. The metabolism of bacterial pathogens has evolved to support virulence in nutrient-limiting host environments. Here, we show that sorbose metabolism promotes the fitness and virulence of *mcr-1-*positive *Escherichia coli* (MCRPEC), but does not affect its resistance to colistin or polymyxin B. Notably, the virulence contribution is also observed in an *mcr-1*-negative background. Genetic disruption of sorbose catabolism (Δ*sorD*) attenuated MCRPEC virulence and fitness both *in vitro* and *in vivo*. Integrated transcriptomic and metabolomic analyses suggest that this attenuation is associated with impaired expression of two major virulence determinants. First, defective sorbose metabolism limits the supply of monosaccharide precursors required for LPS biosynthesis, leading to reduced LPS content. Second, metabolic disruption decreases intracellular cAMP levels, which downregulates *fimH* expression via a cAMP-dependent signaling pathway, thereby compromising bacterial adhesion. Notably, although *sorD* deletion enhances biofilm formation, this increase is insufficient to rescue the *in vivo* virulence defect. Restoration of the sorbose metabolic pathway partially rescues MCRPEC pathogenicity. These findings suggest that sorbose metabolism contributes to MCRPEC pathogenicity by supporting LPS synthesis and regulating *fimH* expression via cAMP signaling. This study indicates a metabolic link between sorbose utilization and MCRPEC pathogenicity, raising the possibility that sorbose, a common food additive, could facilitate MCRPEC pathogenicity.

**IMPORTANCE:**

The spread of colistin-resistant *Escherichia coli* (*E. coli*) limits treatment options for life-threatening infections. This study shows that sorbose metabolism, which utilizes a common dietary sugar, contributes to the fitness and virulence of such resistant bacteria without affecting their colistin resistance. Using *mcr-1*-positive *E. coli*, we find that this metabolic pathway supports lipopolysaccharide synthesis and, via a cAMP-dependent mechanism, promotes bacterial adhesion. Disabling sorbose catabolism attenuates the pathogen in animal models. These findings suggest a previously unrecognized link between a specific carbohydrate metabolism and pathogenesis in drug-resistant *E. coli*, raising the possibility that dietary components may influence infection outcomes.

## INTRODUCTION

The emergence and spread of antimicrobial resistance are major global public health challenges (1). Colistin is the last line of defense antibiotic in the treatment of bacterial infections, but its effectiveness has been limited by the emergence of the mobilized colistin resistance gene, *mcr-1*, which was identified in China in 2016 (2). The main bacterial host of *mcr-1* is *Escherichia coli* (*E. coli*), which raises significant public health and environmental concerns.

Bacterial acquisition of antibiotic resistance and virulence typically incurs high fitness costs (3–5). Clinically, however, *mcr-1-*positive *E. coli* (MCRPEC) simultaneously exhibits drug resistance and retains infective capacity (6, 7). Several recent studies suggest that MCR-positive strains achieve the maintenance of antibiotic resistance and the survival adaptability through metabolic reprogramming (8, 9). However, the mechanism by which they maintain virulence remains unclear.

The evolution of metabolism in bacterial pathogens is intimately linked to their virulence (10–13). During infection, pathogens obtain essential nutrients, including metals, from their host environment. Therefore, alterations in carbon sources (14, 15), amino acids (16), and metal homeostasis (17) are associated with an increased risk of infectious diseases. A previous study showed that pathogen infection may be exacerbated by an increase in glucose metabolism. Sugar kinases at the start of glycolysis modulate the virulence of *Candida albicans* (18). Glucose and glycolysis are also required for successful *Salmonella enterica serovar Typhimurium* infection (19). Enterohemorrhagic *E. coli* co-opts established mechanisms for sensing the metabolites and stress cues in the environment to induce virulence factors in a temporal and energy-efficient manner, culminating in disease (20).

Furthermore, a recent epidemiological study indicates a link between sorbose metabolism and pathogenic potential in MCRPEC, as its detection rate was significantly higher in clinical isolates than in strains from non-infectious sources (21). A study showed that the frequency of L-sorbose utilization differs significantly between pathotypes of *E. coli* and *Shigella* from 93% to 0%. Among the 266 strains of the author tested, this frequency increased in the order *Shigella*, enterotoxigenic *E. coli*, enteroinvasive *E. coli*, *Shiga* toxin-producing *E. coli*, enteroaggregative *E. coli*, enteropathogenic *E. coli*, and neonatal bacterial meningitis *E. coli*. This suggests an association of pathomechanism with the capability to degrade L-sorbose (22).

L-sorbose is commonly found in plants and is regularly used in feed and dietary foods. Bacteria in the *Enterobacteriaceae* family use sorbose as a source of carbon and energy. Bacterial cells employ a sugar-specific phosphoenolpyruvate-dependent phosphotransferase system to transport and phosphorylate the ketohexose to sorbose-1-phosphate. This compound is reduced to D-glucitol-6-phosphate and then oxidized to D-fructose-6-phosphate, which is a metabolite of glycolysis (23). However, the potential contributions of the *sor* operon and sorbose metabolism to bacterial virulence and fitness remain to be elucidated.

Here, we investigated the role of sorbose metabolism in MCRPEC pathophysiology. Our findings suggest that the sorbose catabolic pathway functions as an important virulence determinant that enhances bacterial fitness and pathogenicity, without affecting resistance to colistin and polymyxin B, indicating how metabolic reprogramming drives the pathogenic success of drug-resistant pathogens.

## RESULTS

### Sorbose metabolism augments the virulence of *E. coli*

To investigate the role of sorbose metabolism in the pathogenicity of *E. coli*, we selected a sorbose-utilizing, well-characterized pathogenic model strain: uropathogenic *E. coli* strain CFT073. The *sorD* gene, located on the sorbose (*sor*) operon, encodes a sorbitol-6-phosphate dehydrogenase (Fig. S1A). We constructed *sorD* knockout mutants and the corresponding complemented strains and subsequently generated an MCRPEC strain by introducing a plasmid harboring the *mcr-1* gene into CFT073 (Table S1).

To assess whether the mutant strains retain sorbose metabolic capacity, we cultured them in M9 minimal medium supplemented with 2% sorbose as the sole carbon source. RT-qPCR analysis showed that supplementation with 2% sorbose failed to induce *sorD* expression in either the CFT073Δ*sorD* or CFT073Δ*sorD-mcr-1* strain (Fig. S1B). Consistent with the role of SorD in converting sorbitol to fructose-6-phosphate, disruption of *sorD* led to intracellular accumulation of sorbitol (Fig. S1C). Sorbose metabolism appears not to directly regulate *mcr-1* transcription (Fig. S1D). Functionally, both mutant strains exhibited impaired growth in sorbose-supplemented minimal medium (Fig. S1E), whereas all strains grew comparably under control conditions, including carbon-free M9 medium (Fig. S1F), LB broth (Fig. S1G), and M9 medium supplemented with glucose (Fig. S1H). This metabolic defect was also observed in an independent MCRPEC strain, BE079, where deletion of *sorD* abrogated growth in sorbose-containing medium (Fig. S1I), but not in LB (Fig. S1J). Collectively, these results indicate that disruption of *sorD* abrogates sorbose metabolism in MCRPEC.

**Fig 1:**
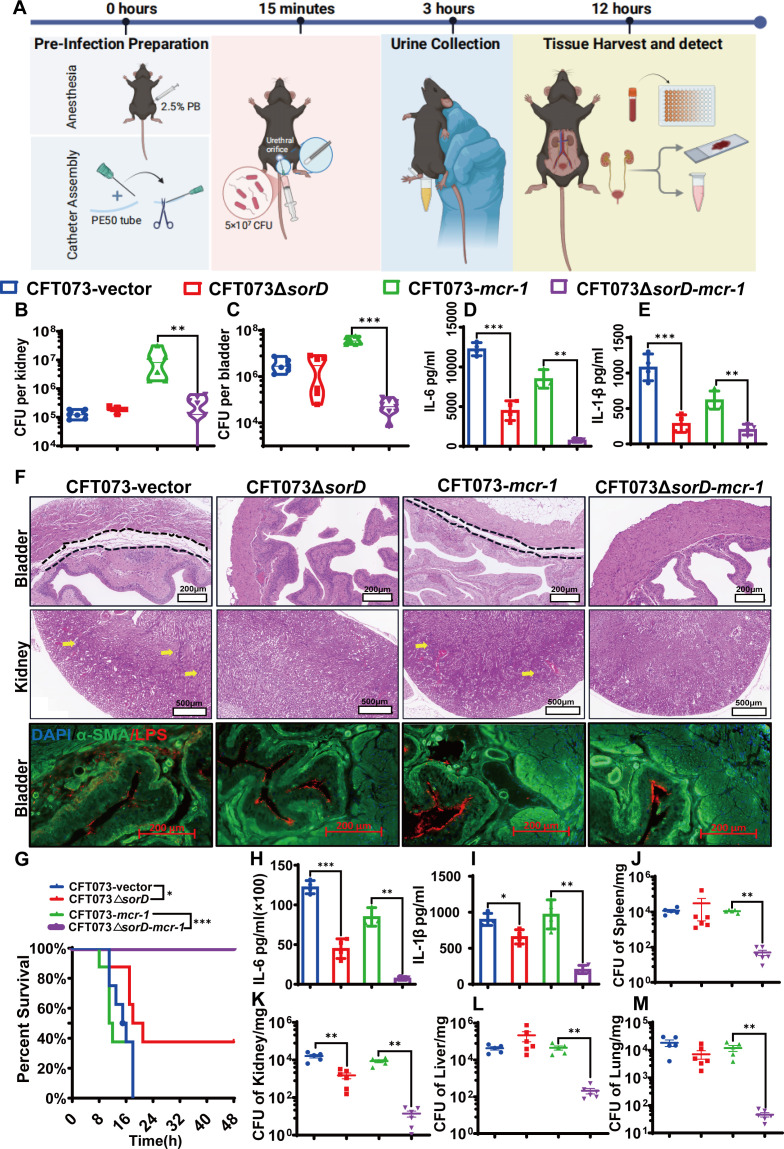
Disruption of the sorbose metabolic pathway impaired *E. coli* pathogenicity. (**A**) Workflow for establishing the mouse UTI model. The illustration was created with Biorender.com. (**B and C**) Bacterial load in the kidney and bladder of mice with urinary tract infection (UTI) (*n* = 6). (**D and E**) Levels of IL-6 and IL-1β in the serum of mice with UTI (*n* = 4). (**F**) Pathological sections of bladder and kidney tissues were prepared. Hematoxylin and eosin (H&E) staining revealed tissue architecture and infiltrating lymphocytes. PMNs are indicated by black arrows, and red blood cells are indicated by blue arrows. Fluorescence staining was used to visualize bacterial adhesion, with green fluorescence indicating α-smooth muscle actin (α-SMA) staining of the cytoskeleton and red fluorescence representing anti-*E*. *coli* LPS staining for the detection of *E. coli*. Scale bar: 200 μm (*n* = 3). (**G**) Survival curves of mice in a model of abdominal infection (*n* = 8). (**H and I**) Levels of IL-6 and IL-1β in the serum of mice with abdominal infection (*n* = 4). (**J through M**) Colony-forming units (CFU) in the lung, liver, spleen, and kidney of mice with abdominal infection (*n* = 5–6). All *n* values represent independent mice. Statistical details are provided in the Materials and Methods. Briefly, data in panels **B through D** and **J through M** were analyzed using Student’s *t*-test (with Welch’s correction for *n* < 5) or the Mann–Whitney *U* test for unequal variances. Survival curves (**G**) were compared using the log-rank (Mantel–Cox) test. Data are presented as mean ± SEM. *P < 0.05, **P < 0.01, ***P < 0.001.

Next, we investigated the effect of impaired sorbose metabolism on virulence in two representative mouse infection models. In a murine urinary tract infection model (Fig. 1A), a higher bacterial load was observed in the kidneys, bladders, and urine of the mice infected with the CFT073-*mcr-1* strain compared to those infected with the CFT073Δ*sorD-mcr-1* strain, whereas no significant difference was observed between CFT073-vector and CFT073Δ*sorD* (Fig. 1B and C and Fig. S2A). Consistent findings were obtained in mice infected with the clinical isolate MCRPEC BE079. Infection with BE079Δ*sorD* resulted in significantly lower bacterial burdens in the mouse bladder and kidneys compared with BE079-vector. No significant difference was observed between BE079Δ*sorD*Δ*mcr-1* and BE079Δ*mcr*-1 (Fig. S2B and C). Mice infected with strains capable of sorbose metabolism exhibited significantly elevated levels of the pro-inflammatory cytokines IL-6 and IL-1β (Fig. 1D and E). Histological examinations of hematoxylin and eosin (H&E)-stained tissue sections revealed that polymorphonuclear cells (PMNs) and red blood cells had infiltrated the mucosal layer of the bladders of mice infected either with the CFT073-vector or CFT073-*mcr-1*. In contrast, bladders infected with the CFT073Δ*sorD-mcr-1* strain exhibited fewer infiltrating immune cells and red blood cells. Additionally, renal tissues from mice infected with sorbose-metabolizing strains showed a higher number of inflammatory cells and bacterial adhesion (Fig. 1F, black arrows). Collectively, these findings show that the capacity to metabolize sorbose may provide a competitive edge to MCRPEC during UTI.

To further investigate the role of sorbose metabolism in systemic infections, we assessed bacterial virulence in a mouse intraperitoneal infection model. After 48 h, all CFT073-vector and CFT073-*mcr-1*-infected mice had died, whereas 40% of CFT073Δ*sorD*-infected mice died. In contrast, all CFT073Δ*sorD-mcr-1*-infected mice survived (Fig. 1G). Likewise, BE079Δ*sorD* caused lower mouse mortality than BE079-vector in the intraperitoneal infection model, with no significant difference between BE079Δ*sorD*Δ*mcr-1* and BE079Δ*mcr-1* (Fig. S2D). Pro-inflammatory cytokines IL-6 and IL-1β levels in the sera of mice infected with CFT073∆*sorD-mcr-1* were significantly decreased compared to those in the CFT073-*mcr-1*-infected group (Fig. 1H and I). Notably, at 14 h post-infection, the mice infected with CFT073∆*sorD-mcr-1* showed significantly lower bacterial loads in their lungs, livers, and kidneys compared to those infected with CFT073-*mcr-1* (Fig. 1J through M). These findings suggest that sorbose metabolism is important for maintaining the pathogenicity of *E. coli* in the host.

### Sorbose metabolism confers a selective survival advantage to MCRPEC by maintaining fitness and membrane integrity

**Fig 2:**
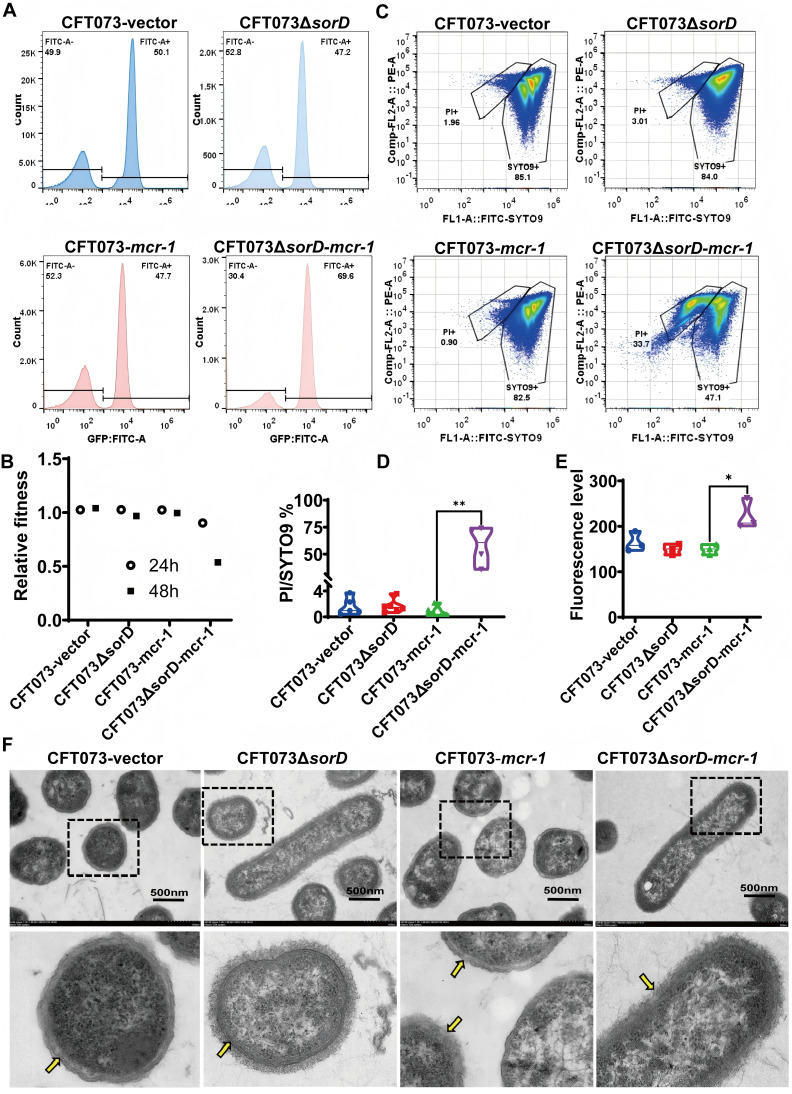
Sorbose metabolic capacity contributed to the survival and growth advantage of CFT073. (**A**) Representative flow cytometry histogram peaks. The positive gate was defined by the reference strain MG1655-GFP. (**B**) Bacterial relative fitness of CFT073 strains in M9 minimal medium was calculated based on the formula provided. (**C**) Representative flow cytometry plot of SYTO9 and propidium iodide (PI) staining, showing the quantification of live and dead bacteria after 48 h of incubation in M9 minimal medium. (**D**) Quantification of live and dead bacteria based on the percentage of SYTO9 and PI staining. Violin plots display the distribution of the data (*n* = 4–6). (**E**) NPN uptake levels used to assess outer membrane permeabilization. Bacteria were cultured in M9 medium supplemented with 2% sorbose prior to the assay (*n* = 3). (**F**) Representative electron microscopy image showed bacterial structures. Prior to sample preparation, bacteria were cultured in M9 with 2% sorbose. Scale bar: 500 nm. All *n* values represent independent experimental replicates. Data in panels **B, D, and E** were analyzed using Student’s *t*-test (with Welch’s correction for *n* < 5) or the Mann–Whitney *U* test for unequal variances. Statistical details are provided in the Materials and Methods. Data are presented as mean ± SEM. *P < 0.05, **P < 0.01.

To examine whether the decreased pathogenicity of Δ*sorD* strains was primarily due to impaired survival advantages, we performed competitive assays in M9 minimal medium. At 24 h, the CFT073-*mcr-1* strain exhibited a slight competitive advantage; however, this advantage had increased approximately twofold after 48 h of coculture (Fig. 2A and B). To further assess viability, we stained the strains grown in M9 minimal medium for 48 h with a live/dead viability kit. Notably, the proportion of dead CFT073Δ*sorD-mcr-1* bacteria was significantly higher than that of live bacteria (>50%; Fig. 2C and D), indicating that disruption of sorbose metabolism severely compromises survival advantage.

Given that MCR-1 expression is known to impair outer membrane (OM) permeability in *E. coli* and *K. pneumoniae*, we hypothesized that the observed fitness defect might be attributable to increased membrane permeability. To test this, we measured OM permeability using the fluorescent probe NPN. The CFT073Δ*sorD-mcr-1* strain generated a more intense fluorescent signal than the CFT073-*mcr-1*, suggesting its increased OM permeability (Fig. 2E). We further examined ultrastructural changes by transmission electron microscopy (TEM). All other strains displayed typical cellular characteristics, including a multilayered cell surface with a well-defined OM, a distinct peptidoglycan layer in the periplasmic space, a regular cytoplasmic membrane, and granular cytoplasm. In contrast, the CFT073Δ*sorD-mcr-1* mutant exhibited marked structural alterations. Its cell wall architecture became indistinct, the peptidoglycan layer was largely absent, and CFT073Δ*sorD* or CFT073Δ*sorD-mcr-1* displayed a capsular structure distinct from that of the CFT073-vector or CFT073-*mcr-1* (Fig. 2F). Notably, despite its significant fitness defect, the CFT073Δ*sorD-mcr-1* mutant remained as resistant to colistin and polymyxin B as the CFT073-*mcr-1* control, with an MIC of 8 μg/mL for both strains.

**Fig 3:**
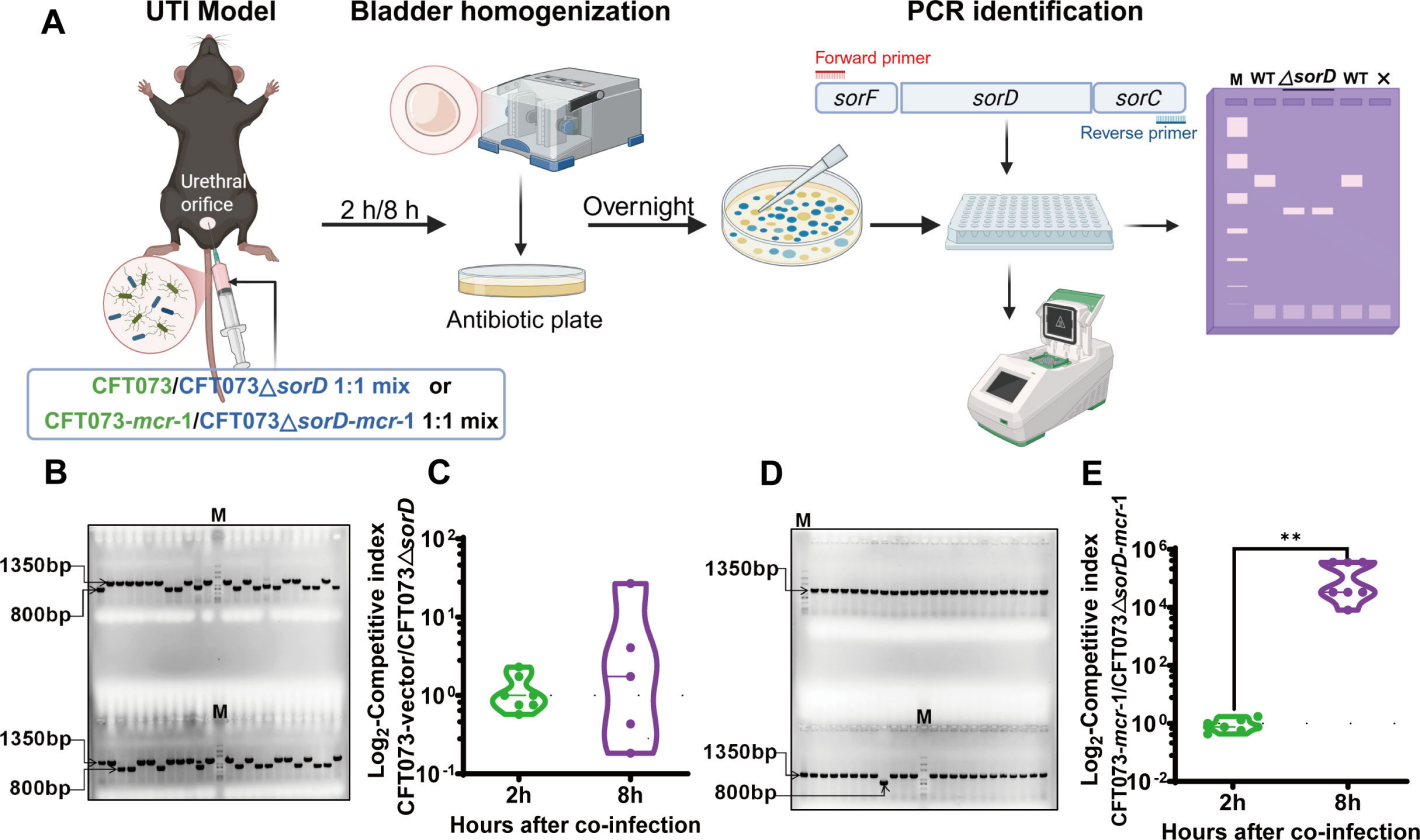
The WT of MCRPEC outcompeted the Δ*sorD* mutant *in vivo*. (**A**) Experimental scheme for assessing competitive bladder colonization. Female C57BL/6 mice were transurethrally instilled with a 1:1 mixture (1 × 10^7^ CFU total) of CFT073/CFT073Δ*sorD* or CFT073-*mcr-1*/CFT073Δ*sorD-mcr-1*. Bladders were harvested at 2 and 8 h post-infection and homogenized. Serial dilutions of the tissue homogenates were plated on LB agar supplemented with 100 μg/mL ampicillin or 4 μg/mL colistin. After overnight incubation, 100 colonies per plate were randomly selected and subjected to genotyping by PCR. The illustration was created with Biorender.com. (**B**) Representative DNA electrophoresis image. The sample from one mouse incubated with CFT073/CFT073Δ*sorD* mixture. (**C**) Competitive index at 2 and 8 h post-infection (*n* = 7). (**D**) Representative DNA electrophoresis image. The sample from one mouse incubated with CFT073-*mcr-1* and CFT073Δ*sorD-mcr-1* mixture. (**E**) Competitive index at 2 and 8 h post-infection (*n* = 7). All *n* values represent independent mice. Data in panels **C and E**) were analyzed using Student’s *t*-test or the Mann–Whitney *U* test for unequal variances. Statistical details are provided in the Materials and Methods. Data are presented as mean ± SEM. **P < 0.01.

To investigate whether the sorbose metabolic pathway confers an advantage for MCRPEC in *vivo*, we co-inoculated mice with a 1:1 mixture of Δ*sorD* mutant and wild-type strains and monitored their relative colonization (Fig. 3A). At 2 h post-infection, the ratio between the two strains was 1:1, whereas at 8 h, the wild-type strain outnumbered the Δ*sorD* strain in the *mcr-1*-positive strains but not in the *mcr*-1-negative background (Fig. 3B through E). These findings suggest that the sorbose catabolic pathway plays a pivotal role in sustaining competitive advantage *in vivo*.

### Sorbose metabolism provides the carbon skeletons and energy to support LPS synthesis and proinflammatory capacity MCRPEC

To examine the impact of sorbose metabolic disruption on the pathogenicity of MCRPEC, we performed integrated transcriptomic and metabolomic profiling on strains cultured under nutrient-restricted conditions (M9 minimal medium supplemented with 2% [wt/vol] sorbose). Multi-omics analysis showed marked transcriptomic and metabolomic differences between CFT073-*mcr-1* and CFT073Δ*sorD-mcr-1* (Table S2).

**Fig 4:**
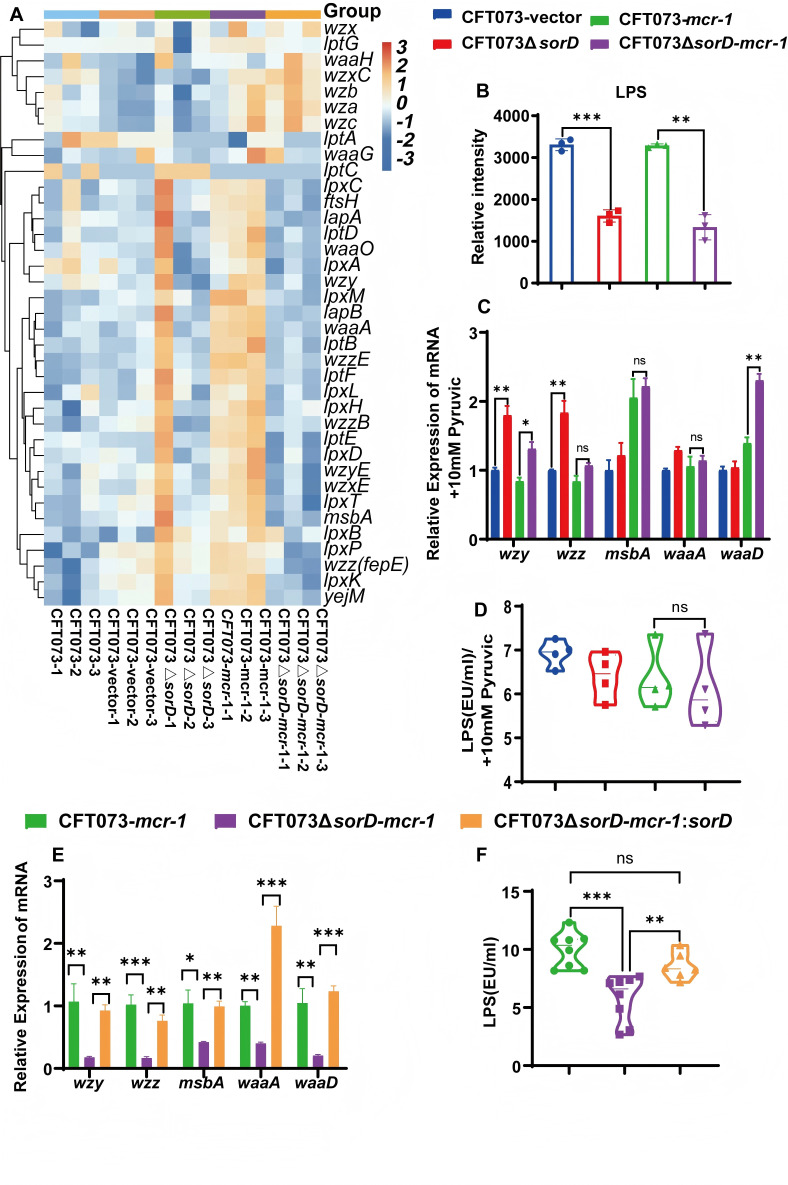
Impaired sorbose metabolism caused defective synthesis of LPS. (**A**) Heatmap showing the expression profiles of LPS biosynthesis-related genes in strains cultured in M9 medium supplemented with 2% sorbose. (**B**) LC-MS analysis of LPS from strains grown in M9 medium supplemented with 2% sorbose (*n* = 3). (**C**) Quantification of LPS oligosaccharide synthesis-related genes using qPCR. Before RNA extraction, the strains were cultivated in M9 minimal medium with 10 mM pyruvate for 48 h (*n* = 4). (**D**) The levels of LPS from the strains cultivated in M9 minimal medium with 10 mM pyruvate for 48 h were measured using a chromogenic endotoxin assay (*n* = 4). (**E**) RT-qPCR analysis of LPS core oligosaccharide biosynthetic gene expression. Strains were cultured in M9 minimal medium supplemented with 2% sorbose for 48 h prior to RNA extraction (*n* = 4). (**F**) The levels of LPS from the strains cultivated in M9 minimal medium with 2% sorbose for 48 h (*n* = 8). All *n* values represent independent experimental replicates. Statistical details are provided in the Materials and Methods. Briefly, data in panels B–D were analyzed using Student’s *t*-test (with Welch’s correction for *n* < 5) or the Mann–Whitney *U* test for unequal variances. Data in panels E–F were analyzed using the Kruskal–Wallis test followed by Dunn’s test with Bonferroni correction (or one-way ANOVA with Tukey’s HSD when parametric assumptions were met). Data are presented as mean ± SEM. *P < 0.05, **P < 0.01, ***P < 0.001, ns, not significant.

Transcriptomics showed that the genes involved in oligosaccharide synthesis, lipid A biosynthesis, and phospholipid binding and transport (*lpt*, *lpx*) were upregulated in CFT073-*mcr-1* strains. In particular, disruption of the sorbose metabolic pathway led to a markedly decreased expression of key genes involved in LPS core oligosaccharide synthesis (*waa*, *wzz*, *wzy*, and *msbA*) in MCRPEC (Fig. 4A). These data suggest that LPS biosynthesis, particularly the synthesis of its key components, including O-antigen and oligosaccharide, was impaired in the sorbose metabolism-deficient mutant.

Given that O-antigen and oligosaccharide are critical structures involved in host immune recognition, we hypothesized that defective LPS biosynthesis may underlie the markedly attenuated pathogenicity of CFT073Δ*sorD-mcr-1*. Accordingly, we explored the relationship between impaired sorbose metabolism and LPS biosynthesis. LC-MS analysis detected a significant reduction in LPS levels in both CFT073Δ*sorD* and CFT073Δ*sorD-mcr-1* strains (Fig. 4B), along with reduced levels of LPS monosaccharide components (Fig. S3A through F). Consistently with this, a commercial LPS quantification kit confirmed that LPS was significantly diminished in the CFT073Δ*sorD-mcr-1* mutant (Fig. S3G).

Given that pyruvate serves as a critical metabolic node in glycolysis and bridges carbohydrate metabolism through multiple biosynthetic pathways, we attempted to rescue LPS biosynthesis through pyruvate supplementation. Supplementation with 10 μM pyruvate rescued LPS biosynthesis in the CFT073Δ*sorD-mcr-1* mutant, restoring core oligosaccharide biosynthetic gene expression (Fig. 4C) and LPS levels (Fig. 4D) to near those of the CFT073-*mcr-1* strain. Similarly, genetic complementation of the *sorD* gene in the CFT073Δ*sorD-mcr-1* restored LPS core oligosaccharide biosynthetic gene expression (Fig. 4E) and, concomitantly, elevated LPS production to the CFT073-*mcr-1* level (Fig. 4F). Taken together, these findings suggest that the sorbose metabolic pathway may contribute to providing sugar precursors for LPS biosynthesis of MCRPEC. Consequently, its disruption attenuates LPS production and, thereby, reduces the bacterium’s proinflammatory capacity.

### Sorbose catabolism promotes MCRPEC epithelial adhesion via sustained *fimH* expression

**Fig 5:**
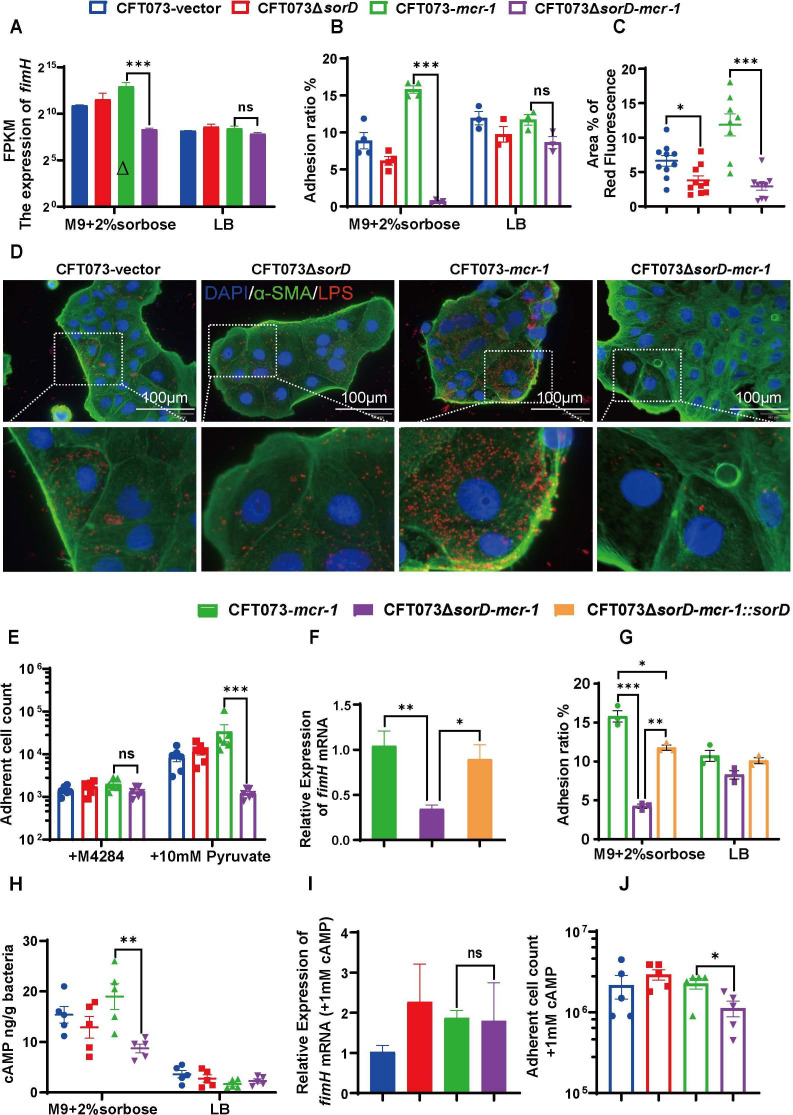
Sorbose metabolism deficiency attenuated MCRPEC virulence through *fimH* downregulation-mediated adhesion impairment. (**A**) RNA-seq analysis of *fimH* mRNA expression level. (**B**) Adhesion ratio of the strains to Caco-2 cells in different media (*n* = 3). (**C**) Quantification of the red fluorescence area ratio (*n* = 10 fields per group). (**D**) Fluorescence microscopy analysis of bacterial adhesion to Caco-2 epithelial cells. Actin (green) and *E. coli* CFT073 (red) were visualized using α-SMA and anti-LPS staining, respectively. Before coculture, the strains were cultivated in M9 minimal medium with 2% sorbose for 48 h. Scale bar: 50 μm. (**E**) Adhesion assay of Caco-2 epithelial cells in the presence of FimH inhibitor M4284 (1 mM) or 10 mM pyruvate (*n* = 6). (**F**) Expression levels of *fimH* mRNA in M9 minimal medium with 2% sorbose were measured using qPCR (*n* = 4). (**G**) Adhesion of strains to Caco-2 cells after growth in LB or M9 minimal medium with 2% sorbose for 48 h (*n* = 6). (**H**) Quantification of intracellular cAMP concentration by ELISA kit (*n* = 5). (**I**) Expression levels of *fimH* mRNA after adding 1 mM exogenous cAMP to the M9 with 2% sorbose medium for 48 h (*n* = 3). (**J**) Adhesion assay after adding 1 mM exogenous cAMP to the culture medium (*n* = 5). All *n* values represent independent experimental replicates or individual microscopic fields. Statistical details are provided in the Materials and Methods. Data in panels** A through C**, **E,** and **H through J** were analyzed using Student’s *t*-test (with Welch’s correction for *n* < 5) or the Mann–Whitney *U* test for unequal variances. Data in panels **F and G** were analyzed using the Kruskal–Wallis test followed by Dunn’s test with Bonferroni correction (or one-way ANOVA with Tukey’s HSD when parametric assumptions were met). Data are presented as mean ± SEM. *P < 0.05, **P < 0.01, ***P < 0.001, ns, not significant.

In both infection models, organ bacterial loads were significantly lower in mice infected with the CFT073Δ*sorD-mcr-1* mutant than in those infected with the CFT073*-mcr-1* strain. We therefore hypothesized that the disruption of sorbose metabolism attenuates bacterial adhesion to host cells. To test this, we examined the expression of adhesin-encoding genes from RNA-seq data. Among the candidates, *fimH*, encoding the key adhesin of type 1 fimbriae in strain CFT073, showed marked downregulation in the Δ*sorD-mcr-1* mutant specifically when grown in M9 medium supplemented with 2% sorbose, while transcript levels were comparable across strains under nutrient-rich conditions (LB broth) (Fig. 5A and Fig. S4A and B). Functionally, both the CFT073Δ*sorD* and CFT073Δ*sorD-mcr-1* strains exhibited significantly reduced adhesion to epithelial cells in a sorbose-dependent manner. Under nutrient-rich conditions, no adherence differences were observed on either Caco-2 or SW 780 cells (Fig. 5B through D and Fig. S4B).

To determine whether *fimH* mediates the adherence defect observed in the Δ*sorD* mutant, we employed the FimH-specific antagonist M4284. The addition of M4284 eliminated the adherence difference between CFT073Δ*sorD* and CFT073Δ*sorD-mcr-1*, whereas supplementation with 10 mM pyruvate (as a carbon source) failed to restore the expected difference (Fig. 5E). Furthermore, genetic ablation of *fimH* in the Δ*sorD* background abolished the adherence disparity when sorbose was the sole carbon source (Fig. S4D). Together, these results indicate that disruption of sorbose metabolism leads to a *fimH*-dependent reduction in adhesion, supporting a metabolic link between sorbose utilization and virulence gene expression in MCRPEC.

To further investigate whether the downregulation of *fimH* and the associated adhesion defect are directly attributable to the loss of *sorD*, we complemented the *sorD* gene in the CFT073Δ*sorD-mcr-1* strain. Complementation restored *fimH* expression and adhesion ratio to levels comparable to those of CFT073-*mcr-1* (Fig. 5F and G), supporting that sorbose metabolism positively regulates *fimH*. Together, these results suggest that *fimH* plays an important role in mediating the effect of the sorbose metabolic pathway on MCRPEC adherence.

We next investigated how sorbose metabolism specifically affects *fimH*. Exogenous pyruvate failed to restore adhesion in the Δ*sorD* mutant (Fig. 5E), suggesting that *fimH* downregulation is not due to general metabolic stress from carbon or energy limitation. Given the role of the cAMP-CRP complex in maintaining bacterial fitness in the absence of glucose, we measured intracellular cAMP concentrations. When sorbose was the sole carbon source, cAMP levels were consistently lower in CFT073Δ*sorD-mcr-1* than in the other strains, whereas all four strains exhibited similarly low cAMP levels in LB (Fig. 5H). Notably, exogenous cAMP restored both *fimH* expression (Fig. 5I) and adherence (Fig. 5J) in CFT073Δ*sorD-mcr-1* to levels observed in CFT073-*mcr-1*. Collectively, these data suggest that disruption of sorbose metabolism impairs FimH-mediated adhesion through a cAMP-dependent regulatory pathway.

### Sorbose metabolism-deficient MCRPEC strains exhibit aggregation as a putative adaptive survival strategy

**Fig 6:**
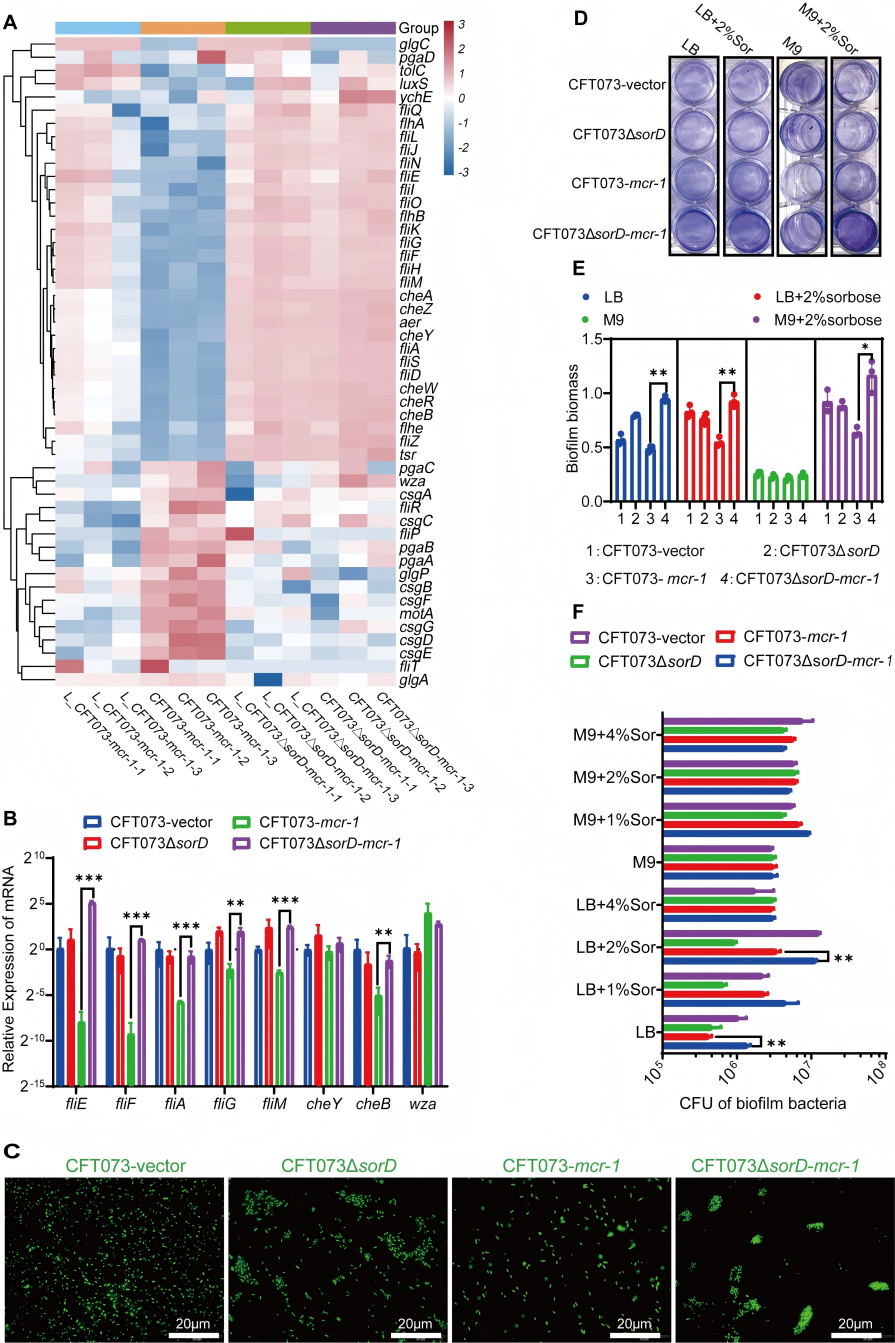
MCRPEC sorbose metabolism-deficient strains tended to aggregate. (**A**) Heatmap showing the expression profiles of bacterial chemotaxis and flagellar assembly-related genes in strains cultured in M9 medium supplemented with 2% sorbose (*n* = 3). (**B**) qPCR analysis of genes involved in chemotaxis and flagellar assembly (*n* = 3). (**C**) Representative fluorescence microscopy images of SYTO9-stained bacteria (green). Scale bar: 20 μm. (**D**) Representative image of crystal violet-stained biofilm formation. (**E**) Quantification of biofilm biomass by measuring the optical density (OD_570_) of crystal violet-stained biofilms in a 24-well plate (*n* = 3). (**F**) Viable bacterial counts (CFU/mL) in biofilms determined using the MBEC assay system (*n* = 3). All *n* values represent independent experimental replicates. Statistical details are provided in the Materials and Methods. Data in panels **B, E, and F** were analyzed using Student’s *t*-test with Welch’s correction or the Mann–Whitney *U* test for unequal variances. Data are presented as mean ± SEM. *P< 0.05, **P < 0.01, ***P < 0.001.

Biofilm formation is a vital survival strategy for bacteria, providing physical protection, coordinating community behavior, and optimizing resource utilization under hostile conditions. Transcriptomic profiling of the CFT073Δ*sorD-mcr-1* strain showed coordinated upregulation of the motility (*fli*) and chemotaxis (*che*) operons (Fig. 6A), consistent with the enhanced aggregation and surface colonization phenotypes. These transcriptional changes were validated by qPCR (Fig. 6B). Functionally, the Δ*sorD* mutant exhibited significant cell aggregation (Fig. 6C), a phenotype that often accompanies enhanced biofilm formation. Consistent with this, the Δ*sorD-mcr-1* strain showed a substantially enhanced capacity to form biofilms on abiotic surfaces when cultured in sorbose-containing medium (Fig. 6D and E). Moreover, the viable cell counts within CFT073Δ*sorD-mcr-1* biofilm significantly exceeded those of CFT073-*mcr-1* (Fig. 6F). Together, these results suggest that disruption of sorbose metabolism may trigger a global adaptive response in MCRPEC, potentially promoting biofilm formation as a survival mechanism under nutrient stress.

### Reconstitution of the sorbose metabolic pathway restores MCRPEC pathogenicity

To further test the contribution of sorbose metabolism to MCRPEC virulence, we performed genetic complementation by reintroducing the *sorD* gene into the CFT073Δ*sorD-mcr-1* strain via pBAD24 without induction. Functional characterization showed that the complemented strain, CFT073Δ*sorD-mcr-1 :: sorD*, exhibited partial recovery of sorbose catabolic activity when grown in sorbose-supplemented medium (Fig. S5A). RT-qPCR analysis revealed that *sorD* transcription was restored to 89.6% of the CFT073-*mcr-1* level (Fig. S5B).

**Fig 7:**
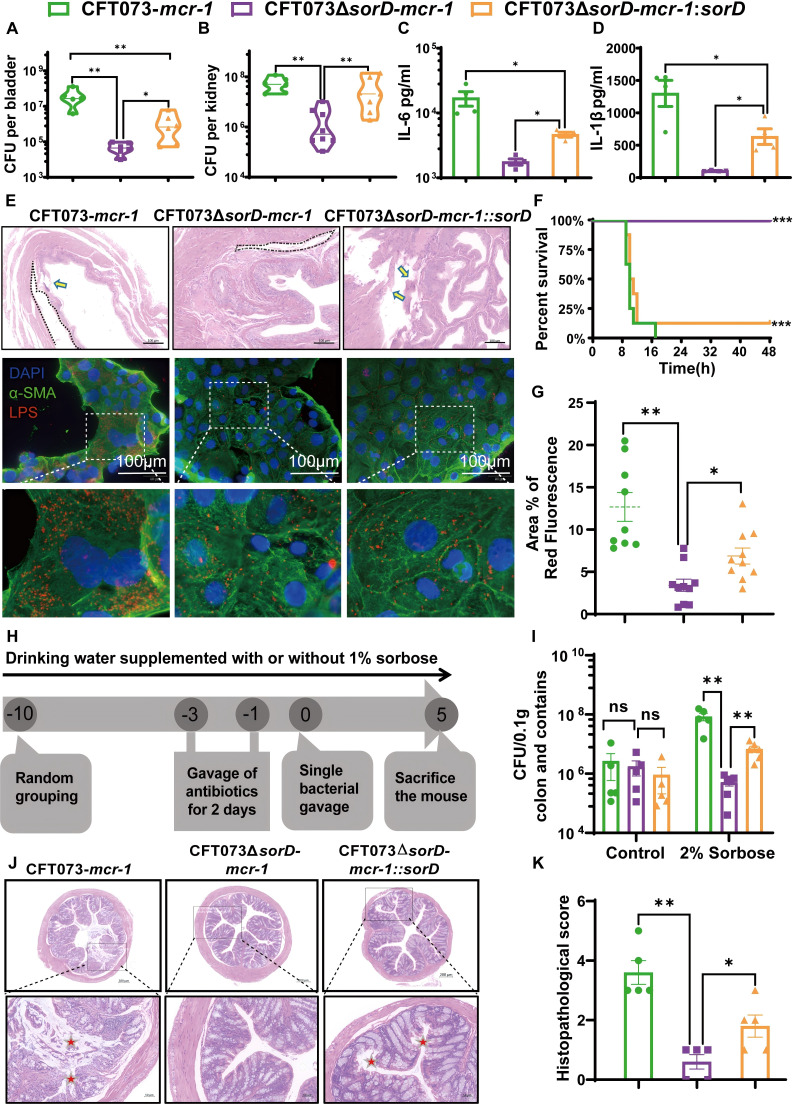
Restoration of the sorbose metabolic pathway restored MCRPEC pathogenicity. (**A and B**) Bacterial load (CFU) in the bladder and kidney of mice with UTI (*n* = 5–6). (**C and D**) Serum IL-6 and IL-1β levels of UTI mice (*n* = 4). (**E**) Top: Representative histopathological sections of the bladder from the UTI model mouse. Bottom: Representative fluorescence microscopy image of bacterial adhesion to Caco-2 epithelial cells. Green: α-SMA (actin); red: LPS-stained *E. coli* CFT073; blue: DAPI (nuclei). Scale bar: 50 μm. (*n* = 3). (**F**) Survival analysis of mice in an abdominal infection model (*n* = 8). (**G**) Quantification of the red fluorescence area ratio. (**H**) Timeline of the intestinal colonization experiment. Beginning 10 days before bacterial challenge, SPF C57BL/6 mice received 1% (wt/vol) sorbose in drinking water. A broad-spectrum antibiotic cocktail was administered during the last 2–3 days to deplete the indigenous microbiota. On day 0, mice were gavaged with 5 × 10^7^ CFU of the indicated strain. Animals were euthanized on day 5 post-gavage bacteria for colonic tissue. (**I**) Intestinal colonization capacity measured by CFU enumeration per 0.1 g colon and contains (*n* = 5). (**J**) Representative H&E staining of histological sections of mouse colons. (**K**) The histological score was used to assess the tissue damage and inflammation in the colon sections (*n* = 5). All *n* values represent independent mice. Data in panels **A through D, G, I, and K** were analyzed by Kruskal–Wallis test followed by Dunn’s test with Bonferroni correction (or one-way ANOVA with Tukey’s HSD when parametric assumptions were met). Survival curves (**F**) were compared using the log-rank (Mantel–Cox) test. Data are presented as mean ± SEM. *P < 0.05, **P < 0.01, ***P < 0.001, ns, not significant.

In the UTI model, the complemented strain regained significant pathogenic potential, with bacterial burdens in murine bladder and kidney tissues reaching levels comparable to those induced by the CFT073-*mcr-1* strain and significantly exceeding those caused by the *sorD*-deficient mutant strain (Fig. 7A and B). The complemented strain induced a moderate host inflammatory response, with systemic proinflammatory cytokine levels being significantly elevated compared to those observed after CFT073Δ*sorD-mcr-1* infection, yet remaining below those elicited by the CFT073-*mcr-1* strain (Fig. 7C and D). Histopathological examination showed that the complemented strain (CFT073Δ*sorD-mcr-1 :: sorD*) induced substantial bladder tissue damage, characterized by mucosal erosion and extensive epithelial detachment, similar to that observed with the CFT073-*mcr-1* strain (Fig. 7E, yellow arrows). This intermediate virulence phenotype was further observed in the sepsis model, where the complemented strain displayed mortality kinetics comparable to those of CFT073-*mcr-1* during the acute phase of the disease (0–24 h post-infection), while final mortality rates remained intermediate between those induced by CFT073-*mcr-1* and CFT073Δ*sorD-mcr-1* (Fig. 7F). Importantly, genetic complementation partially restored MCRPEC adhesion to epithelial cells (Fig. 7G), suggesting that sorbose metabolism is essential for both initial host cell attachment and subsequent pathogenesis.

To assess intestinal colonization capacity, we monitored bacterial persistence in drinking water supplemented with or without 2% sorbose (Fig. 7H). Differences in intestinal colonization were exclusively observed in mice whose drinking water was supplemented with sorbose (Fig. 7I). Colons from CFT073-*mcr-1*-infected mice exhibited pronounced disruption of mucosal architecture and marked lymphocytic infiltration, whereas CFT073Δ*sorD-mcr-1*-infected mice showed almost no such histopathological changes. Infection with the CFT073Δ*sorD-mcr-1::sorD* complemented strain resulted in only mild mucosal lesions (Fig. 7J and K). These results suggest that the sorbose metabolic pathway is important for sustaining MCRPEC infection and intestinal colonization.

## DISCUSSION

Metabolic reprogramming is a pivotal strategy employed by bacteria to enhance their prevalence and pathogenicity (24–26). The *sor* operon is significantly enriched among clinical MCRPEC isolates, prompting us to investigate whether sorbose metabolism contributes to fitness and virulence in this resistant background. We show that the sorbose operon (*sor*) functions as an important virulence determinant without affecting colistin/polymyxin B resistance. Importantly, the virulence contribution is general: it occurs in both *mcr-1*-positive and *mcr-1*-negative strains. However, the fitness effect of sorbose metabolism is background-specific: Δ*sorD* impairs fitness only in the *mcr-1*-positive strain, while it is unaffected in the *mcr-1*-negative background under the conditions tested. These findings exemplify how metabolic reprogramming drives virulence evolution, suggesting that pathogenicity can emerge not only through acquisition of canonical virulence factors but also through metabolic adaptations that reshape the fitness landscape of *E. coli*.

The mechanistic basis for this fitness advantage may involve the sophisticated coupling of sorbose metabolism to the production of two pivotal virulence factors: LPS and FimH adhesin. On the one hand, sorbose-derived carbon skeletons and energy may serve as building blocks for the robust synthesis of LPS (Fig. 4). This is particularly critical for *mcr-1*-bearing strains, as the MCR-1-mediated LPS modification can affect membrane homeostasis (27, 28). Efficient sorbose catabolism might therefore alleviate this burden, ensuring optimal LPS assembly, membrane integrity, and innate immune evasion.

The sustained expression of FimH, which is essential for adhesion to host epithelial cells, indicates a metabolic checkpoint for colonization. It is plausible that sorbose metabolism sustains the intracellular energy pool or signals the upregulation of type 1 fimbriae, thereby supporting a firm foothold in the host (Fig. 5). This dual support system could represent an evolutionary adaptation in which the assimilation of a specific carbohydrate, whether derived from a host or a diet, may have been co-opted to power the pathogen’s invasive apparatus. This may help explain how MCRPEC maintains a high transmission potential in the presence of *mcr-1* plasmid.

In addition to pathway-specific effects, we observed that the Δ*sorD* mutant exhibited a significant shift toward a biofilm-associated state rather than a planktonic state (Fig. 6). This may represent a metabolic attenuation strategy that enhances bacterial survival and persistence. A previous study showed that disruptions in the sorbose pathway led to depleted UDP-glucose pools and sedoheptulose-7-phosphate levels, and that these compounds are key precursors for LPS biosynthesis (29). Additionally, trehalose metabolism can influence virulence in some bacterial species, from enabling growth and modulating the immune response to infection (30). These observations suggest that multiple metabolic bottlenecks may contribute to the attenuated virulence of the Δ*sorD* mutant through compromised colonization, endotoxin production, and lifestyle changes.

Importantly, several issues remain unresolved. First, this investigation was conducted using *E. coli*, the primary host of *mcr-1* plasmid, and the generalizability of these findings to other bacterial hosts remains to be determined. Second, the evolutionary mechanisms through which MCRPEC acquired the sorbose-metabolizing *sor* operon remain to be defined. Third, the molecular basis by which the sorbose catabolic pathway governs the biosynthesis of LPS monosaccharide moieties and modulates FimH expression has yet to be elucidated. In this regard, the Δ*sorD* mutant exhibits increased membrane permeability, altered envelope ultrastructure, reduced viability, and enhanced biofilm formation; therefore, we cannot fully exclude that part of the observed *in vivo* attenuation may involve general stress responses. Nonetheless, the specific rescue of LPS levels by pyruvate and the restoration of *fimH* expression/adhesion by exogenous cAMP support a role for LPS biosynthesis and cAMP-dependent *fimH* regulation. In the complementation experiments, however, the complemented strain displayed *sorD* expression at 89.6% of the wild-type level. The native *sor* promoter is efficiently induced by sorbose, whereas the plasmid-based pBAD plasmid cannot be activated under the same conditions and only supports basal expression. This reduced expression may result in the partial phenotypic rescue. Thus, while the current data suggest pathway-specific contributions, we cannot exclude the possibility that broader stress-related mechanisms also play a role in the observed fitness and virulence defects. Finally, whether the *sor* operon possesses additional functions beyond sorbose catabolism and the modulation of virulence determinants described herein remains to be explored.

**Fig 8:**
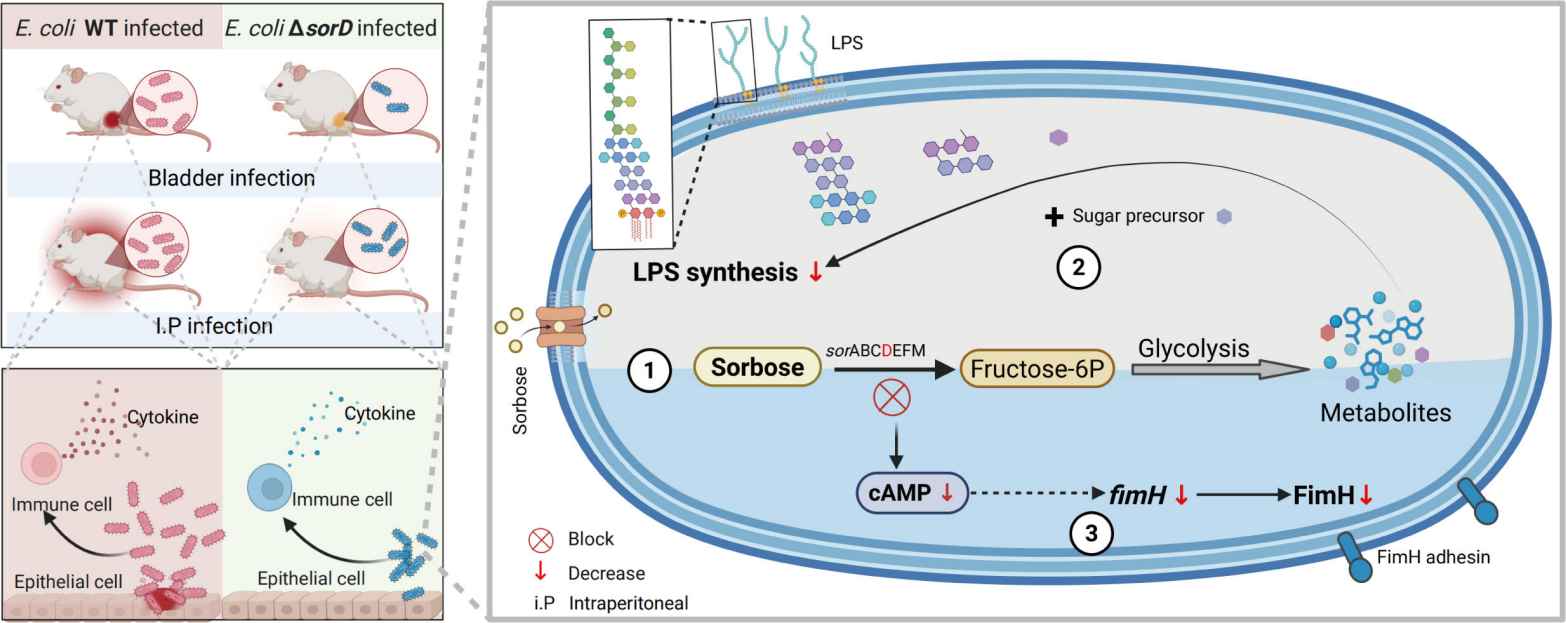
Schematic model of sorbose metabolism-mediated pathogenicity in MCRPEC. Disruption of sorbose catabolism (Δ*sorD*) leads to two major metabolic consequences. First, it reduces the availability of monosaccharide precursors required for LPS biosynthesis, thereby impairing LPS production. Second, it decreases intracellular cAMP levels, which in turn downregulates *fimH* expression through a pathway that remains to be elucidated. These two parallel routes compromise bacterial fitness and virulence in MCRPEC. “WT” represents both the CFT073-vector and CFT073-*mcr-1* strain. Similarly, “Δ*sorD*” represents the CFT073Δ*sorD* and CFT073Δ*sorD-mcr-1* strain. The illustration was created with Biorender.com.

In summary, our study indicates that the prevalent carriage of the *sor* operon in clinical MCRPEC is of functional importance. Our data suggest that sorbose metabolism supports fitness and augments virulence in MCRPEC, potentially by contributing to LPS and maintaining *fimH*-mediated adhesion (Fig. 8). This work thus points to a link between sorbose metabolism and pathogenicity in *E. coli*. Beyond the mechanistic insight, our findings raise the possibility that dietary sorbose, a widely used food additive, could influence the intestinal persistence of MCRPEC. We note that antibiotic pretreatment and sorbose supplementation limit the extrapolation of the gut colonization results to natural intestinal ecology.

## MATERIALS AND METHODS

### Strain construction

The Δ*sorD* and Δ*fimH* mutant strains were constructed as previously described (27). The pREDCas9 plasmid was first electrotransformed into the CFT073 strain. Subsequently, the single-guide RNAs (sgRNAs) designed via the CRISPOR web server (https://crispor.gi.ucsc.edu/crispor.py) were annealed to form double-stranded DNA fragments, which were then inserted into the spacer region of the pgRNA (addgene #44251) plasmid. To construct the donor DNA, two 500 bp homologous arms were amplified separately and fused together with fusion PCR. The pgRNA plasmid and the donor DNA were further electrotransformed into the CFT073 strain that had been pre-transformed with the pREDCas9 plasmid. Positive transformants were screened using antibiotic-resistant agar plates, and the positive clones were finally verified by PCR and Sanger sequencing.

To generate pACYC-NP-*mcr-1*, the *mcr-1* gene and its native promoter were amplified from an *mcr-1*-carrying IncX4 plasmid and cloned into pACYDuet-1, a low-copy number plasmid (containing the p15A origin of replication). CFT073-*mcr-1* and the corresponding control strain were generated by electroporating either a pACYC-NP-*mcr-1* plasmid or an empty pACYC-NP vector into CFT073.

To generate pBAD24-*sorD*, the *sorD* gene, together with its native promoter, was amplified from the CFT073 genome and inserted into the pBAD24 plasmid.

To generate pBAD24-*fimH*, the *fimH* gene, together with its native promoter, was amplified from the CFT073 genome and inserted into the pBAD24 plasmid.

All strains and plasmids used in this study are listed in Table S1. All primers are listed in Table S3.

### Bacterial and cell growth conditions

CFT073 (ATCC 700928), MG1655, and ATCC 25922 were obtained from ATCC. BE079 was previously isolated from the urine of a patient with urinary tract infection. All strains were primarily grown in Luria-Bertani (LB) broth or on LB agar plates at 37°C. When indicating nutrition restriction, the strains were grown overnight on LB and then diluted 1:10 and grown to stationary phase in M9 minimal medium with 2% (wt/vol) sorbose (Sigma, CA). The *sorD* and *fimH* complementation strains were cultured with appropriate antibiotics and without arabinose induction.

Caco-2, SW780, and THP-1 cells were obtained from iCell. Caco-2 cells were cultured in DMEM medium supplemented with 10% fetal calf serum (Procell China). SW780 cells were cultured in L15 medium supplemented with 10% fetal calf serum (Procell China). All cells were cultured in a humidified cell culture incubator supplemented with 5% CO_2_ at 37°C.

### Sorbitol quantification

Bacterial sorbitol was quantified using a quantification kit (Solarbio, China) following the manufacturer’s instructions. The CFT073 strains were cultured in nutrient-limited medium for 48 h. A bacterial suspension of 10 mL was collected and concentrated to a concentration of 1 × 10^9^ colony-forming units (CFU)/mL. Then, 5 mL of the bacterial culture was collected by centrifugation, and the resulting precipitate was resuspended in 500 μL of distilled water and homogenized using a grinding rod. The homogenate was boiled in a metal bath for 10 min, cooled to room temperature, and centrifuged at 8,000 × *g* for 10 min. The resulting supernatant was collected for measurement.

### Growth curve assay

*E. coli* strains were inoculated in LB or M9 minimal medium with varying concentrations of sorbose. The strains were then seeded into 96-well plates at a concentration of 10^6^ cells per well in quadruplicate and incubated at 37°C in a microplate reader (BioTek Epoch2, CA). The absorbance values, or an optical density at 600 nm (OD_600_), for each well were recorded every 30 min for 48–72 h.

### Mouse model

#### Urinary tract infection

Uropathogens were evaluated in an optimal environment using a murine model of urinary tract infection (UTI). Female C57BL/6 mice (8–10 weeks old) were procured from Sun Yat-Sen University, and all animal experiments were conducted in accordance with the guidelines of the SYSU Animal Care and Use Committee (SYSU-IACUC-2024-B1504). The mice were kept in a temperature-controlled room with a 12 h light/dark cycle and ambient humidity. *E. coli* strain CFT073 was introduced into the urinary tract of mice, as previously described (31). Briefly, bacteria were grown, washed, and diluted with sterile 0.9% NaCl to a concentration of 1 × 10^9^ cells/mL, and 50 μL of this bacterial suspension was added to a 1 mL syringe for a final concentration of 5 × 10^7^ CFU/mouse. A sterile PE50 urinary catheter (REWARD, China) was connected to the syringe using scissors; approximately 1–2 mm of the needle tip was covered with the catheter. To perform the procedure, the catheter was first immersed in lubricating jelly. Each mouse was then anesthetized with 2.5% avertin (Sigma-Aldrich), and the lubricated catheter was slowly inserted into the urethra until it was at approximately 3–4 mm from the opening. Bacteria were slowly instilled through this urinary catheter. Urine samples were collected 3 h after infection to preliminarily assess the success of the model based on urine CFU, and mice were sacrificed to obtain blood, bladder, and kidney samples 12 h after infection. Each experimental group consisted of at least three mice.

#### Peritonitis-sepsis Infection

A mouse model was used to assess the virulence of the strains in a peritonitis-sepsis infection. Female BALB/c mice aged 6–8 weeks were infected with CFT073 at a sublethal dose of 10^8^ CFU per mouse via intraperitoneal injection. The survival rate of the mice was monitored after 6 h of infection and analyzed using survival curves. To accurately assess the bacterial load of the mice organs, a new group of mice were confidently injected with 5 × 10^7^ CFU per mouse. The CFU counts were then confidently determined in the blood, lung, liver, spleen, and kidney 12 h after infection.

#### Competition assays *in vivo*

Competition assay was performed following a previously described protocol, with slight modifications (32). UTI model was employed to evaluate the colonization advantage between wild-type and Δ*sorD* CFT073 strains. The genotype of each isolate was assessed using PCR. Primer pairs targeting the two flanking regions of the *sorD* locus were listed in Table S3. A competitive index of 1 (100%) indicates that each strain is at equal levels.

#### Gut colonization

Ten days prior to the administration of the colonization procedure, 1% sorbose was added to the drinking water of all mice. To ablate both the gut microbiome and the tumor microbiome, 8-week-old mice were administered an antibiotic cocktail (ATBx) as described with some modifications (33). Three days prior to the commencement of the colonization process, the mice were administered an antibiotic cocktail via oral gavage for a period of two consecutive days. The antibiotic mixture comprised kanamycin (0.4 g/L), gentamicin (0.035 g/L), colistin (0.057 g/L), metronidazole (0.215 g/L), and vancomycin (0.045 g/L). Subsequently, an oral gavage was administered, containing 5 × 10^7^ CFU of bacteria. On the fifth day following bacterial administration, the mice were euthanized. The colon and its contents were meticulously collected, subsequently homogenized, and then serially diluted. Subsequently, the dilutions were plated onto LB agar plates containing 4 μg/mL colistin.

### Enzyme-linked immunosorbent assay (ELISA)

The levels of IL-6, IL-1β, and TNF-α in the serum of infected mice were quantified using a commercially available ELISA kit (Neobioscience, China), following the manufacturer’s instructions.

### Hematoxylin and eosin stain (H&E)

The tissue was fixed overnight in 4% (vol/vol) paraformaldehyde. Subsequently, the tissues were dehydrated and embedded in paraffin, following the previously established protocol (34). The paraffin blocks were sectioned at a thickness of 4.5 μm, and the resulting sections were stained with H&E.

### Dead/live bacterial cell fluorescence analysis

The LIVE/DEAD BacLight Bacterial Viability and Counting Kit (ThermoFisher, CA) was used to determine the ratio of live to dead bacteria after culturing in nutrient-restricted media. The bacteria were stained and collected following the manufacturer’s instructions. Single-color controls were prepared for instrument adjustment prior to the first experiment. Flow cytometry (Cytoflex, Beckman) was used for the analysis of the stained bacteria. After adjustment of the flow cytometer, the control or experimental samples containing stained bacteria and microspheres were applied to ensure that both populations were on scale. The raw data were analyzed using FlowJo 10.0.

### Outer membrane permeability

Bacterial growth conditions were as described above. Grown bacteria were washed twice and collected by centrifugation at 5,000 × *g* for 5 min. They were then diluted to 10^9^ CFU/mL with phosphate-buffered solution (PBS). Then, 100 μL of the bacterial suspension was added to a 96-well plate. The bacteria were stained with a final concentration of 10 mM fluorescent probe 1-N-phenylnaphthylamine (NPN) (Sigma-Aldrich, St. Louis, MO, USA). The test substance and NPN buffer were pipetted onto the plates beforehand, and the bacterial suspension was added immediately before fluorescence measurement. Results were expressed as relative fluorescence units (35).

### Transmission electron microscope (TEM)

CFT073 strains were cultured in nutrient-restricted media for 48 h. The supernatant was removed by centrifugation at 10,000 × *g* for 5 min. The resulting pellet was fixed with 2.5% glutaraldehyde at 25°C for 30 min, followed by 24 h at 4°C. The resulting pellet was rinsed with PBS (pH 7.4), post-fixed with 1% OsO₄ (4°C, 2 h), and then washed with PBS. Pellets were dehydrated with gradient ethanol (30%–100%, 15 min each; 100% ethanol twice), infiltrated with ethanol-epoxy resin (1:1, 2 h) and pure epoxy resin (overnight), then embedded and polymerized at 60°C for 48 h. Ultrathin sections (60–80 nm) were cut, mounted on copper grids, and stained with 2% uranyl acetate (15 min) and lead citrate (5 min). The sample was observed and imaged with TEM (HITACHI, Japan).

### Competition assays *in vitro*

To determine the relative fitness of CFT073, CFT073Δ*sorD*, CFT073-*mcr-1*, and CFT073Δ*sorD-mcr-1*, competition experiments were conducted. These strains were pitted against a GFP-labeled *E. coli* MG1655 carrying plasmid pgRNA-J23115-superGFP for constitutive expression. The proportional changes in the strains during competition were measured using flow cytometry, as previously described (28).

After culturing the bacteria overnight, the cell density was normalized to an OD_600_ of 1.0. The bacteria were diluted 1:10 in M9 media and mixed with GFP-labeled MG1655 at a 1:1 ratio. The initial proportion of GFP and non-GFP cells was determined using flow cytometry (Cytoflex, Beckman) prior to the start of the competition. After 24 and 48 h of competition, the bacterial populations were analyzed using FlowJo software to determine the proportion of GFP-positive cells relative to the unlabeled cells. The relative fitness of the competing strains was calculated using a precise formula.

### Minimum inhibitory concentration (MIC) determination

The minimum inhibitory concentrations (MICs) of colistin and polymyxin B were determined by the broth microdilution method according to the guidelines of the Clinical and Laboratory Standards Institute (CLSI) document M100. Briefly, bacterial strains were cultured overnight in Mueller-Hinton broth and adjusted to a final inoculum of approximately 5 × 10⁵ CFU/mL. Two-fold serial dilutions of colistin and polymyxin B (ranging from 0.125 to 64 μg/mL) were prepared in 96-well microtiter plates. After inoculation, plates were incubated at 37°C for 16–20 h. The MIC was defined as the lowest concentration of antibiotic that completely inhibited visible bacterial growth. *E. coli* ATCC 25922 was included as a quality control strain in each independent experiment. All assays were performed in triplicate with at least three independent biological replicates.

### RT-qPCR

Total RNA was extracted from bacteria or cells using the SteadyPure General RNA Extraction Kit (AG China). After completion of the extraction process, the RNA concentration was measured using a Nanodrop microspectrophotometer, and any genomic DNA was removed using gDNA Eraser. The RNA was then reverse-transcribed into cDNA using random primers and the PrimeScript RT Master Mix kit (TAKARA, Japan), following the manufacturer’s instructions. Quantitative PCR (qPCR) was performed on a CFX 96 Real-Time PCR Detection System (Bio-Rad) using TB Green Premix Ex Taq (TAKARA, Japan) in 20 μL volumes. Expression changes were calculated using the ΔΔCt method, with normalization to a housekeeping gene and a control condition.

### Sample preparation for RNA sequencing

For bacteria RNA sequencing, the CFT073 strains were grown to mid-logarithmic phase in LB (OD_600_ = 0.6–0.7) or M9 minimal media containing 2% (wt/vol) sorbose (OD_600_ = 0.3–0.4). Bacteria were harvested and centrifuged at 4℃ 2,000 × *g* for 10 min. Bacterial pellets were snap-frozen in liquid nitrogen and stored at −80°C until RNA extraction using TRIzol (Invirogen), according to the manufacturer’s instructions. RNA quality was checked using a Nanodrop One spectrophotometer (ThermoFisher Scientific, Waltham, MA, USA). The method of library construction was described in our previously published article (36).

### Sample preparation for liquid chromatography-mass spectrometry (LC-MS) analysis

Sampling was performed as described for RNA sequencing. LC–MS analysis was performed using the method described in our previous study (36). Briefly, intracellular metabolites were extracted by adding 500 μL of a solution (methanol: water = 4:1, vol/vol) containing an internal standard to the samples, followed by vortexing for 3 min. Next, the samples were immersed in liquid nitrogen for 5 min, followed by placement on dry ice for another 5 min. The samples underwent a rigorous freeze-thaw process, being thawed on ice, and vortexed for 2 min, which was repeated three times to ensure optimal results. After centrifugation at 12,000 rpm for 10 minutes at 4°C, a 200 μL sample of the supernatant was transferred for liquid chromatography-mass spectrometry analysis.

### Bacterial adhesion assays

To perform the adhesion assay, 4 × 10^4^ Caco-2 or SW780 cells were seeded in two 24-well plates and grown overnight without antibiotics. The bacteria were cultured as previously described, washed twice with PBS, and resuspended at a concentration of approximately 10^8^ CFU/mL (OD_600_ = 0.5). Then, 80 μL of the bacterial suspension was added to the Caco-2 cells, resulting in a MOI of 20. After 3 h, one of the two 24-well plates was used to fix the cells and view adhesion formation through fluorescence microscopy. The other plate was used to quantify adherent bacteria on the cells by counting the number of CFU per well. To remove non-adherent bacteria cells, the cells were washed three times with 1 mL of PBS and then lysed with 1 mL of Triton-X solution (0.3% [vol/vol] in PBS). The bacterial cell solution was serially diluted and plated on LB agar. The resulting CFUs were then counted. Finally, the CFUs were counted.

### Immunofluorescence microscopy

Caco-2 cells were fixed with 4% paraformaldehyde for 10 min at 4°C, permeabilized with 0.1% Triton X-100 for 15 min, and blocked with 1% BSA for 1 h, all prepared in PBS. We then incubated primary and secondary IgG antibodies (Abcam), including rabbit anti-α-SMA, mouse anti-*E*. *coli* LPS, Dylight 594-conjugated anti-mouse, and Alexa 488-conjugated anti-rabbit antibodies, at room temperature for 1 h. Finally, bacterial and mammalian DNA were stained with DAPI (Thermo, CA) for 10 min, performing three washes with PBS between each step. The coverslips were mounted with fluorescence mounting medium (ThermoFisher CA) and examined using an orthogonal fluorescence microscope (Olympus Corporation BX63, Japan).

### LPS isolation and quantification

The LPS of CFT073 was extracted using an LPS extraction kit (iNtRON Biotechnology, UK), following the manufacturer’s instructions. Prior to the extraction steps, we performed treatment with protease K to eliminate protein contamination. We added Protease K (AppliChem, Germany) at a concentration of 20 mg/mL to the cell mixture and incubated it at 55°C for 30 min. The LPS concentration was measured using the Pierce Chromogenic Endotoxin Quant Kit (ThermoFisher CA), following the manufacturer’s instructions.

### Biofilm detection and live bacteria count

500 μL 10^7^/ml CFU CFT073 strains were seeded in each well of a 24-well plate and inoculated at 37℃ for 72 h. The wells were then washed with PBS, and the biofilms were fixed with a 4% paraformaldehyde for 10 min and stained with 0.1% crystal violet for 30 min. Subsequently, the biofilms were photographed and then eluted with 75% ethyl alcohol, and the absorbance was measured at 570 nm using a microplate reader (BioTek Epoch2, CA).

The number of live cells in biofilm was evaluated in a MBEC Assay Biofilm Inoculator (Innovotech, Canada). Biofilm formed on the pegs of the MBEC Assay Biofilm Inoculator when planktonic bacteria adhere to the surface. Approximately 10^5^ bacteria were seeded into each well of rows of a fresh 96-well plate and inoculated for 24–48 h at 37℃. A rinse plate was prepared by filling each well of a sterile 96-well plate with 200 µL of sterile saline. After removing the MBEC Assay Biofilm Inoculator from the incubator following biofilm formation, loosely attached cells were removed by placing the lid into the rinse plate for approximately 10 s. Following the rinse step, the plate was transferred to the sonicator and sonicated on high for 30 min to dislodge the biofilm. Serial dilutions were then prepared and spot-plated for CFU determination.

### Replication and data statistics

The replication of each experiment is detailed in the figure legends. The reported *n* for each figure panel represents the number of biological replicates. Data were analyzed and visualized using GraphPad Prism v8. Normality was assessed using the Shapiro–Wilk test; equality of variances using Levene’s test. For comparisons involving only two independent pre-planned groups (e.g., Δ*sorD* vs vector), Mann–Whitney *U* tests (or *t*-tests when assumptions were met) were applied, with no correction for multiple comparisons across unrelated comparisons. For three groups requiring all pairwise comparisons (e.g., *-mcr-1*, Δ*sorD-mcr-1*, complemented), the Kruskal–Wallis test followed by Dunn’s test with Bonferroni correction was performed; when parametric assumptions were met, one-way ANOVA with Tukey’s HSD was used. Kaplan–Meier survival curves were compared using the log-rank (Mantel–Cox) test. A *P*-value of less than 0.05 was considered statistically significant. For RNA-seq, differential expression was analyzed with DESeq2 (|log₂ fold change| > 1, FDR < 0.05, Benjamini–Hochberg). For metabolomics, XCMS was used for peak picking and alignment, followed by KEGG enrichment (hypergeometric test, *P* < 0.05). All data presented in the figures are represented as means ± SEM. At least three biological replicates were conducted for each experiment. *P*-values are indicated as follows: *P < 0.05, **P < 0.01, ***P < 0.001.

## Data Availability

The transcriptomic and metabolomics sequencing data are available from the NCBI Gene Expression Omnibus under the accession number PRJNA1310473.

## SUPPLEMENTAL MATERIAL

Supplemental Material msphere.00191-26-s0001.pdf Tables S1 to S3; Fig. S1 to S5.

ASM does not own the copyrights to Supplemental Material that may be linked to, or accessed through, an article. The authors have granted ASM a non-exclusive, world-wide license to publish the Supplemental Material files. Please contact the corresponding author directly for reuse.
