# Supplementary material for: Sorbose metabolism promotes fitness and virulence in Escherichia coli
Source: mSphere. 2026 Jun 15;11(7):e00191-26. doi: 10.1128/msphere.00191-26 (PMC13410766; doi:10.1128/msphere.00191-26)
Supplement: Supplemental Material — Tables S1 to S3; Fig. S1 to S5. [file msphere.00191-26-s0001.pdf]

Table S1. Strains used in this study.

Strains and plasmids	Description	Source or reference
DH5α	Commercialised competence cells for plasmid replication	AlpalifeBio
CFT073	Uropathogenic *Escherichia coli*	ATCC
CFT073-vector	MCR-1 negative *sorD* wild-type strain	This study
CFT073Δ*sorD*	MCR-1 negative *sorD* mutant strain	This study
CFT073-*mcr-1*	MCR-1 positive strain	This study
CFT073Δ*sorD-mcr-1*	MCR-1 positive and *sorD* mutant strain	This study
CFT073Δ*sorD*-*mcr*-1:*sorD*	Carrying complement *sorD gene*	This study
CFT073Δ*fimH*	MCR-1 negative *fimH* mutant strain	This study
CFT073Δ*fimH-mcr-1*	MCR-1 positive and *fimH* mutant strain	This study
BE079	From the urine of a patient with urinary tract infection. Carry *sor* operon and *mcr-1* plasmid	Isolated previouly from clinical
BE079Δ*sorD*	MCR-1 positive and *sorD* mutant strain	This study
BE079Δ*mcr-1*	MCR-1 negative strain	This study
BE079Δ*sorD*Δ*mcr-1*	MCR-1 negative *sorD* mutant strain	This study
MG1655	As a reference for competition experiments	ATCC
MG1655-sfgfp	Express GFP fluorescent green, as a reference for competition experiments	This study
pREDcas9	For CRISPR–Cas9-mediated genome editing	Lab stock
pgRNA-J23115-superGFP	sgRNA vector	Lab stock
pACYCDuet-NP-698-MCR-1	Vector for *mcr-1* protein expression in *E. coil*	Lab stock
pBAD24-*sorD*	expression vector, encodes the *sorD* gene	This study
pBAD24-*fimH*	expression vector, encodes the *fimH* gene	This study

Table S2-1 Differential expression results of metabolites

Group: CFT073△*sorD-mcr-1* vs. CFT073-*mcr-1*								
**Kegg_pathway**	**ko_ID**	**Sig_compound**	**compound**	**Sig_compound_all**	**compound_all**	**IndexList**	**CIDList**	**Pathway**
Purine metabolism	ko00230	7	18	53	322	MEDP0150;ME	C05512+C001	https:\\www.genome.jp\dbget-bin\www_bget?map00230
Metabolic pathways	ko01100	30	177	53	322	MEDP0150;ME	C05512+C026	https:\\www.genome.jp\dbget-bin\www_bget?map01100
Nucleotide metabolism	ko01232	8	18	53	322	MEDP0150;ME	C05512+C001	https:\\www.genome.jp\dbget-bin\www_bget?map01232
ABC transporters	ko02010	28	129	53	322	MEDP0150;ME	C05512+C012	https:\\www.genome.jp\dbget-bin\www_bget?map02010
Phenylalanine, tyrosine and tryptophan biosy	ko00400	1	7	53	322	MEDN0833*19	C02637	https:\\www.genome.jp\dbget-bin\www_bget?map00400
Biosynthesis of various plant secondary meta	ko00999	2	11	53	322	MEDN0833*19	C02637+C001	https:\\www.genome.jp\dbget-bin\www_bget?map00999
Biosynthesis of siderophore group nonriboso	ko01053	1	3	53	322	MEDN0833*19	C02637	https:\\www.genome.jp\dbget-bin\www_bget?map01053
Biosynthesis of secondary metabolites	ko01110	8	66	53	322	MEDN0833*19	C02637+C001	https:\\www.genome.jp\dbget-bin\www_bget?map01110
Biosynthesis of amino acids	ko01230	3	25	53	322	MEDN0833*19	C02637+C009	https:\\www.genome.jp\dbget-bin\www_bget?map01230
Biosynthesis of cofactors	ko01240	6	26	53	322	MEDN0833*19	C02637+C012	https:\\www.genome.jp\dbget-bin\www_bget?map01240
Thiamine metabolism	ko00730	1	3	53	322	MEDP2364	C01279	https:\\www.genome.jp\dbget-bin\www_bget?map00730
Toluene degradation	ko00623	1	6	53	322	MEDN2028	C06677	https:\\www.genome.jp\dbget-bin\www_bget?map00623
Microbial metabolism in diverse environment	ko01120	5	47	53	322	MEDN2028;ME	C06677+C009	https:\\www.genome.jp\dbget-bin\www_bget?map01120
Degradation of aromatic compounds	ko01220	1	13	53	322	MEDN2028	C06677	https:\\www.genome.jp\dbget-bin\www_bget?map01220
Arginine and proline metabolism	ko00330	4	13	53	322	MEDP1741;ME	C00179+C033	https:\\www.genome.jp\dbget-bin\www_bget?map00330
Glycine, serine and threonine metabolism	ko00260	1	4	53	322	MEDP0039	C00719	https:\\www.genome.jp\dbget-bin\www_bget?map00260
Inositol phosphate metabolism	ko00562	2	2	53	322	MEDN1224;ME	C19799+C000	https:\\www.genome.jp\dbget-bin\www_bget?map00562
Biofilm formation - Escherichia coli	ko02026	1	1	53	322	MEDN0421	C00575	https:\\www.genome.jp\dbget-bin\www_bget?map02026
Bacterial chemotaxis	ko02030	17	88	53	322	MEDN1910;ME	C00107+C001	https:\\www.genome.jp\dbget-bin\www_bget?map02030
Pyrimidine metabolism	ko00240	4	14	53	322	MEDN1612;ME	C02354+C000	https:\\www.genome.jp\dbget-bin\www_bget?map00240
Starch and sucrose metabolism	ko00500	1	3	53	322	MEDN0227*04	C00092	https:\\www.genome.jp\dbget-bin\www_bget?map00500
Streptomycin biosynthesis	ko00521	1	2	53	322	MEDN0227*04	C00092	https:\\www.genome.jp\dbget-bin\www_bget?map00521
Two-component system	ko02020	1	3	53	322	MEDN0227*04	C00092	https:\\www.genome.jp\dbget-bin\www_bget?map02020
Phosphotransferase system (PTS)	ko02060	2	12	53	322	MEDN0227*04	C00092+C002	https:\\www.genome.jp\dbget-bin\www_bget?map02060
Fructose and mannose metabolism	ko00051	1	7	53	322	MEDN1221*04	C00275	https:\\www.genome.jp\dbget-bin\www_bget?map00051
Amino sugar and nucleotide sugar metabolis	ko00520	1	11	53	322	MEDN1221*04	C00275	https:\\www.genome.jp\dbget-bin\www_bget?map00520
O-Antigen nucleotide sugar biosynthesis	ko00541	8	10	53	322	MEDN1221*04	C00275	https:\\www.genome.jp\dbget-bin\www_bget?map00541
Biosynthesis of nucleotide sugars	ko01250	1	10	53	322	MEDN1221*04	C00275	https:\\www.genome.jp\dbget-bin\www_bget?map01250

Ascorbate and aldarate metabolism	ko00053	1	5	53	322	MEDN0240	C01040	https:\\www.genome.jp\dbget-bin\www_bget?map00053
C5-Branched dibasic acid metabolism	ko00660	1	7	53	322	MEDN0701*008	C00490	https:\\www.genome.jp\dbget-bin\www_bget?map00660
Lysine biosynthesis	ko00300	1	3	53	322	MEDP0430*123	C00956	https:\\www.genome.jp\dbget-bin\www_bget?map00300
Lysine degradation	ko00310	1	8	53	322	MEDP0430*123	C00956	https:\\www.genome.jp\dbget-bin\www_bget?map00310
2-Oxocarboxylic acid metabolism	ko01210	2	12	53	322	MEDP0430*123	C00956+C004	https:\\www.genome.jp\dbget-bin\www_bget?map01210
Valine, leucine and isoleucine degradation	ko00280	1	8	53	322	MEDP1144	C00407	https:\\www.genome.jp\dbget-bin\www_bget?map00280
Valine, leucine and isoleucine biosynthesis	ko00290	1	5	53	322	MEDP1144	C00407	https:\\www.genome.jp\dbget-bin\www_bget?map00290
Cyanoamino acid metabolism	ko00460	1	4	53	322	MEDP1144	C00407	https:\\www.genome.jp\dbget-bin\www_bget?map00460
Aminoacyl-tRNA biosynthesis	ko00970	1	9	53	322	MEDP1144	C00407	https:\\www.genome.jp\dbget-bin\www_bget?map00970
Histidine metabolism	ko00340	1	7	53	322	MEDP0089	C05135	https:\\www.genome.jp\dbget-bin\www_bget?map00340
Methane metabolism	ko00680	1	3	53	322	MEDP0878*123	C01046	https:\\www.genome.jp\dbget-bin\www_bget?map00680
D-Amino acid metabolism	ko00470	1	12	53	322	MEDP2478*244	C05620	https:\\www.genome.jp\dbget-bin\www_bget?map00470
Pantothenate and CoA biosynthesis	ko00770	1	4	53	322	MEDN0249	C05944	https:\\www.genome.jp\dbget-bin\www_bget?map00770
Porphyrin metabolism	ko00860	1	2	53	322	MEDN0622	C00931	https:\\www.genome.jp\dbget-bin\www_bget?map00860
Sphingolipid metabolism	ko00600	1	1	53	322	MEDP0871	C00836	https:\\www.genome.jp\dbget-bin\www_bget?map00600
Pinene, camphor and geraniol degradation	ko00907	1	1	53	322	MEDP0716	C01499	https:\\www.genome.jp\dbget-bin\www_bget?map00907

Table S2-2 Differential expression results of transcripts

Group: CFT073△*sorD-mcr-1* vs. CFT073-*mcr-1*					
ID	Description	GeneR	BgRatio	pvalue	p.adjust
ko02035	Bacterial motility proteins [BR:ko02035]	23/106	72/2261	1.5012768	9.60817211961166e-
ko00543	Exopolysaccharide biosynthesis	7/106	13/2261	5.5678047	1.78169752303431e-
ko02020	Two-component system	19/106	128/226	3.7072987	7.90890390033442e-
ko01503	Cationic antimicrobial peptide (CAMP) resista	7/106	24/2261	7.3068011	0.0011690881762242
ko00630	Glyoxylate and dicarboxylate metabolism	8/106	33/2261	9.3544081	0.001197364238875[illegible]
ko02040	Flagellar assembly	9/106	45/2261	0.0001675	0.001787446452725[illegible]
ko00020	Citrate cycle (TCA cycle)	7/106	31/2261	0.0004226	0.003399999113364[illegible]
ko02030	Bacterial chemotaxis	5/106	15/2261	0.0004249	0.003399999113364[illegible]
ko00785	Lipoic acid metabolism	4/106	13/2261	0.0023518	0.016724416252096[illegible]
ko00520	Amino sugar and nucleotide sugar metabolism	6/106	35/2261	0.0049227	0.031505418584764[illegible]
ko05133	Pertussis	4/106	17/2261	0.0067699	0.039388686630994[illegible]
ko02024	Quorum sensing	7/106	52/2261	0.0095358	0.050857619407484[illegible]
ko00907	Pinene, camphor and geraniol degradation	2/106	4/2261	0.0122803	0.057369374154289[illegible]
ko00071	Fatty acid degradation	3/106	11/2261	0.0125495	0.057369374154289[illegible]
ko01003	Glycosyltransferases [BR:ko01003]	3/106	14/2261	0.0249898	0.106623453131844
ko00362	Benzoate degradation	2/106	6/2261	0.0288619	0.112000716634618
ko02000	Transporters [BR:ko02000]	31/106	481/226	0.0297501	0.112000716634618
ko02010	ABC transporters	11/106	127/226	0.0325658	0.115789596819353
ko00930	Caprolactam degradation	1/106	1/2261	0.0468819	0.15791801485137
ko00280	Valine, leucine and isoleucine degradation	2/106	8/2261	0.0506724	0.162151852131038
ko00720	Carbon fixation pathways in prokaryotes	4/106	32/2261	0.0597798	0.182186141090566
ko00260	Glycine, serine and threonine metabolism	4/106	34/2261	0.0718385	0.208984920486438
ko00380	Tryptophan metabolism	2/106	10/2261	0.0766326	0.213238551807452
ko01501	beta-Lactam resistance	2/106	11/2261	0.0908732	0.234459339394819
ko00642	Ethylbenzene degradation	1/106	2/2261	0.0915856	0.234459339394819
ko00910	Nitrogen metabolism	3/106	26/2261	0.1193699	0.280602312780419
ko02025	Biofilm formation - Pseudomonas aeruginosa	2/106	13/2261	0.1213591	0.280602312780419
ko02022	Two-component system [BR:ko02022]	4/106	41/2261	0.1227635	0.280602312780419
ko00592	alpha-Linolenic acid metabolism	1/106	3/2261	0.1342115	0.286318045491015
ko00997	Biosynthesis of various other secondary meta	1/106	3/2261	0.1342115	0.286318045491015
ko02044	Secretion system [BR:ko02044]	8/106	112/226	0.1506854	0.311092548606686
ko00650	Butanoate metabolism	3/106	30/2261	0.1634567	0.326913524806775
ko05134	Legionellosis	1/106	4/2261	0.1748553	0.339113361474353
ko00541	O-Antigen nucleotide sugar biosynthesis	2/106	17/2261	0.1879208	0.353733293560044
ko00340	Histidine metabolism	1/106	6/2261	0.2505575	0.443064144071398
ko00360	Phenylalanine metabolism	1/106	6/2261	0.2505575	0.443064144071398
ko00051	Fructose and mannose metabolism	3/106	38/2261	0.2623345	0.443064144071398
ko01001	Protein kinases [BR:ko01001]	2/106	22/2261	0.2759722	0.443064144071398
ko05111	Biofilm formation - Vibrio cholerae	2/106	22/2261	0.2759722	0.443064144071398
ko03110	Chaperones and folding catalysts [BR:ko0311	4/106	57/2261	0.2769150	0.443064144071398
ko02026	Biofilm formation - Escherichia coli	3/106	44/2261	0.3407152	0.523008487207263
ko00410	beta-Alanine metabolism	1/106	9/2261	0.3513963	0.523008487207263
ko01504	Antimicrobial resistance genes [BR:ko01504]	1/106	9/2261	0.3513963	0.523008487207263
ko00640	Propanoate metabolism	2/106	29/2261	0.3978499	0.578690777272645
ko00540	Lipopolysaccharide biosynthesis	2/106	31/2261	0.4311045	0.585037139895937
ko00310	Lysine degradation	1/106	12/2261	0.4387778	0.585037139895937

ko00660	C5-Branched dibasic acid metabolism	1/106	12/2261	0.4387778	0.585037139895937
ko00670	One carbon pool by folate	1/106	12/2261	0.4387778	0.585037139895937
ko01005	Lipopolysaccharide biosynthesis proteins [BR:	2/106	34/2261	0.4791177	0.620643576936295
ko00470	D-Amino acid metabolism	1/106	14/2261	0.4904454	0.620643576936295
ko00040	Pentose and glucuronate interconversions	2/106	35/2261	0.4945753	0.620643576936295
ko00400	Phenylalanine, tyrosine and tryptophan biosyn	1/106	17/2261	0.5592394	0.688294690470243
ko00190	Oxidative phosphorylation	2/106	42/2261	0.5943797	0.717741637032953
ko00860	Porphyrin metabolism	1/106	21/2261	0.6368513	0.754786729797799
ko03021	Transcription machinery [BR:ko03021]	1/106	23/2261	0.6704148	0.780119086776661
ko00620	Pyruvate metabolism	2/106	52/2261	0.7105432	0.812049401274101
ko00564	Glycerophospholipid metabolism	1/106	28/2261	0.7414725	0.831718411125819
ko00920	Sulfur metabolism	1/106	29/2261	0.7537448	0.831718411125819
ko03000	Transcription factors [BR:ko03000]	5/106	139/226	0.7936191	0.860763835641953
ko02060	Phosphotransferase system (PTS)	1/106	34/2261	0.8069660	0.860763835641953
ko00270	Cysteine and methionine metabolism	1/106	37/2261	0.8332500	0.874229570422397
ko01007	Amino acid related enzymes [BR:ko01007]	1/106	42/2261	0.8694058	0.89745121126538
ko00230	Purine metabolism	1/106	67/2261	0.9618485	0.968708542175592
ko03009	Ribosome biogenesis [BR:ko03009]	1/106	71/2261	0.9687085	0.968708542175592

Table S3. PCR primers used in this study.

Primer	Sequence	Description
sorD-up homo-5F	GTAATGGGATCATGCAGTCTCAG	For upstream homology arm
sorD-up homo-3R	ATGCGCCACCGGTGACAATA	
sorD-do homo-5F	TGCGCACGCCGGAATATGAAGAA	For downstream homology arm
sorD-do homo-3R	TTATCAACCAGTTCGAGCGCCAGC	
sgRNA-5F	TAGCCAGATGGTCGATATTCACGG	Guided Cas9 for targeted editing of DNA
sgRNA-3R	AAACCCGTGAATATCGACCATCTG	
mcr-1-5F	ATGATGCAGCATACTTCTGTG	Complete amplification of the MCR-1 gene
mcr-1-3R	TCAGCGGATGAATGCGGT	
sorD TTA-5F	TTAGCCGCGCGTTTTG	Amplify the *sorD* gene and insert in the plasmid to generate the complement strain
sorD ACG-3R	GCAAACGTGGTTAAATTTGC	
fimH-up homo-5F	CGTTCGCAAGTGCGACGATAC	For upstream homology arm
fimH-up homo-3R	CAGCAAACAGGGTAATAACTCGTT	
fimH-do homo-5F	AGTTATTACCCTGTTTGCTGGTGACTGCAG GGAATGTGCAATC	For downstream homology arm
fimH-do homo-3R	TGATCCAGCAACCGGTCAGCT	
fimH-sgRNA-5F	CCAATGGTACCGCAATCCCT	Guided Cas9 for targeted editing of fimH gene
fimH-sgRNA-3R	AGGGATTGCGGTACCATTGG	
gyrA-5F	ACTGCCGTCGATGGTATCCAG	As a bacterial reference gene in qPCR
gyrA-3R	CGGTGCAGGTAGATGACTGCGA	
sorM-2-5F	AACAGGGCGAAAATCAGC	qPCR quantification of CFT073 sorbose operon RNA expression
sorM-2-3R	GCAGAACATCCTCGACCA	
sorC-5F	ACGCAGTGCACCAACAAT	
sorC-3R	GGCGCAATGGTTGAAACG	
sorE-5F	GGCTGGAATTTGTGCTGC	
sorE-3R	GACCTTCCTGAAATGCAG	
sorA-5F	ATGTAGAGCAGGGCCATG	
sorA-3R	TTGTGACCCGTGGACTG	
sorB-5F	TCTGACGCTACGACGCG	
sorB-3R	CCCACAGGATGCCTTAGC	
sorF-5F	AATCCTCCTCCGCTGT	
sorF-3R	TTACAGTGCGGCAGCC	
sorD-5F	CTGCTAATCTTCCGGC	
sorD-3R	GCGCTCAATAGCTTCACG	
lpxC-5F	ACGTATCGTTCAGGCGACG	qPCR quantification of CFT073 mRNA expression
lpxC-3R	ACCGGTGGATTCAAGTCGGT	
waaA-5F	TGCTTTACACCGCCCTTCTCT	
waaA-3R	AGCGGATGGCGGTAAAAACC	
lptA-5F	GCATTTGCCGTAACCGGAGA	
lptA-3R	GACGGGTAACGACCACTTTG	
lptC-5F	AGCCAGACGTTGGGTTATCATT	
lptC-3R	GGGTTATAGACGAGCGTGTCC	
fimH-5F	ACCCTGTTTGCTGTACTGCTGA	
fimH-3R	CGACCAGGTTTTGCCCCACAT	
fliM-5F	ACGAACCGACAGCCAGTGT	
fliM-3R	TGGTACGGCTGAATGCGGAT	
fliG-5F	AGTGCAAACCCTGAGCGCT	

fliG-3R	GCACGTTCTTCACCCAGAGCT	
fliA-5F	ACCGCGCCAATCTCTTTGAG	
fliA-3R	ATGAGTGGCGCGAAGAGCA	
fliE-5F	CCACCAGCTTATTACGCACCT	
fliE-3R	CGCTCGATCGCATAAGCGATA	
fliF-5F	TGAGTGGCTTAATCGCCTGCG	
fliF-3R	GACAATTGCGCCACCATCCT	
cheB-5F	ATATTGATGGCCTCGCGCG	
cheB-3R	CCATCGGCCATCGGTAGATGT	
cheY-5F	TTCACCACATAGCCACTGGCC	
cheY-3R	CTCCGACTGGAACATGCCCAAT	
wza-5F	GTCGCTGGCTGTATCCGTC	
wza-3R	AGCTGGATATGTCCGATGCG	
GAPDH-5F	GGAGCGAGATCCCTCCAAAAT	As a human reference gene in qPCR
GAPDH-3R	GGCTGTTGTCATACTTCTCATGG	

Extended Data

Figure S1

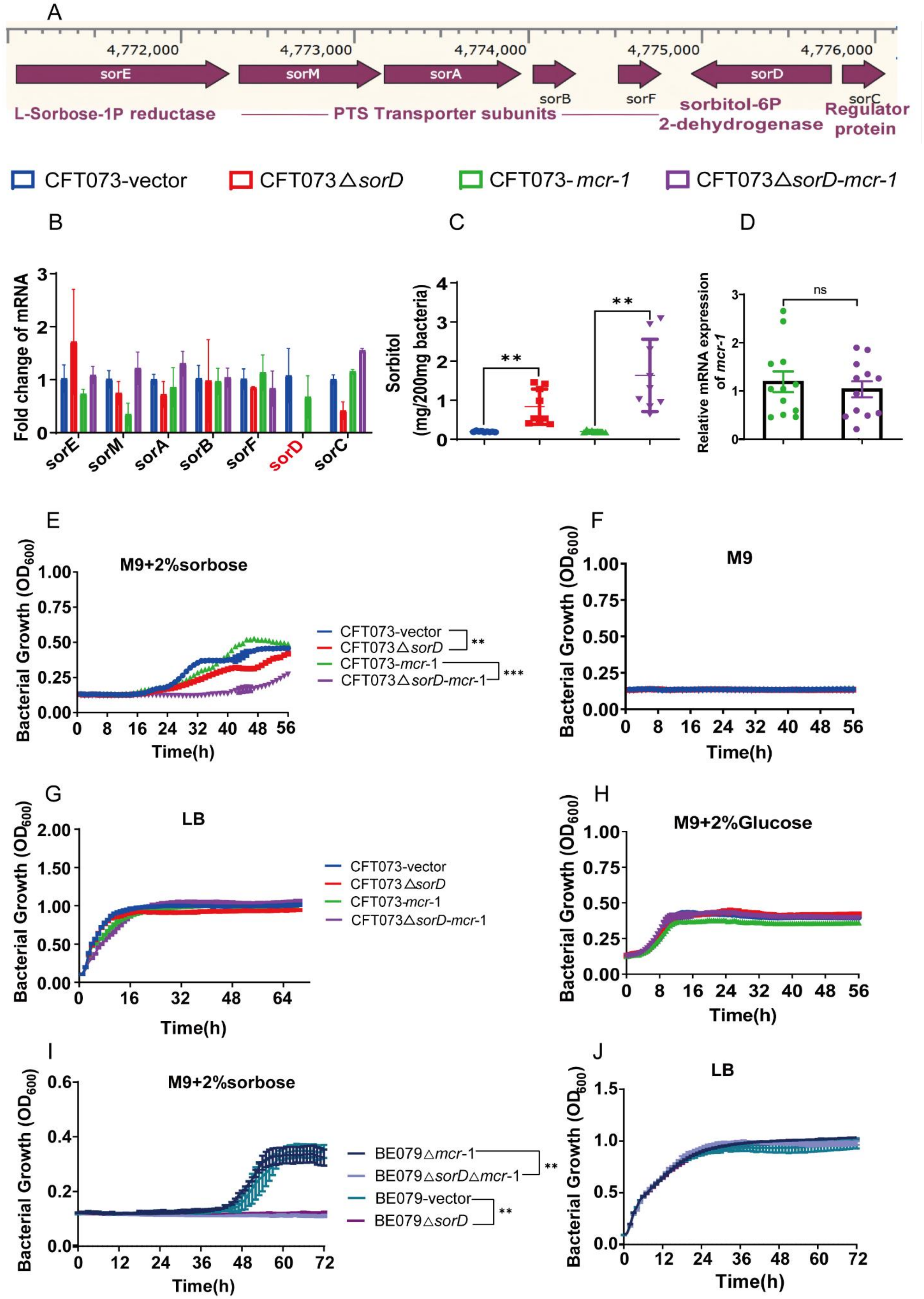

Figure S1. Sorbose metabolism and growth of *E. coli* strains with or without *sorD gene*. (A) Schematic representation of the sorbose utilization locus in the *E. coli* strain CFT073. (B) Absence of *sorD* gene expression induction by 2% sorbose in minimal medium. Data are presented as mean ± SEM (n = 3). (C) Quantification of intracellular sorbitol using a commercial kit. Data are presented as mean ± SEM (n = 7–9). (D) RT-qPCR analysis of *mcr-1* expression (n = 12). (E) Growth curve of the strain in nutrient-limited medium. Each data point represents an individual replicate (n = 3). (F–H) Growth curve of the strain in nutrient-limited or nutrient-rich medium. Each data point represents an individual replicate (n = 3). All 'n' values represent independent experimental replicates. Data in (C, D) were analysed by Student's *t*-test (with Welch's correction for n < 5) or the Mann–Whitney U test for unequal variances. Data in (E–J) were analysed by a two-way ANOVA with Dunnett's. Data are presented as mean ± SEM. *p < 0.05, **p < 0.01, ***p < 0.001.

Figure S2

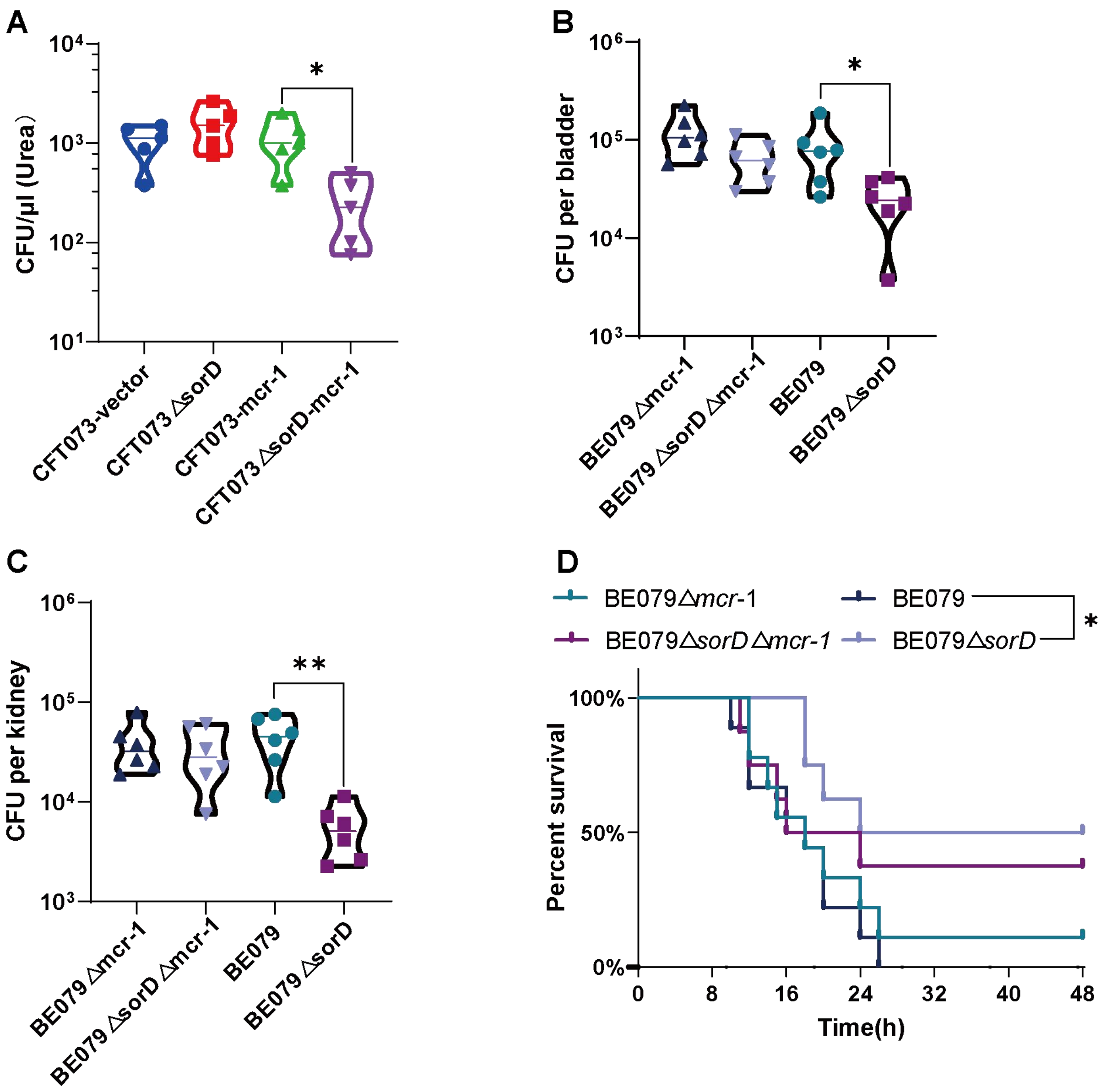

Figure S2. Disruption of the sorbose metabolic pathway impaired MCRPEC pathogenicity. (A) Bacterial load in the urine of mice with CFT073 strains induced-UTI (n = 5). (B–C) Bacterial load in the kidney and bladder of mice with BE079 strains infection (n = 6). (D) Survival curves of mice in a model of abdominal infection (n = 8). All 'n' values represent independent mice. Data in (A–C) were analysed by Student's *t*-test or the Mann–Whitney U test for unequal variances. Survival curves (D) were compared using the log-rank (Mantel-Cox) test. Data are presented as mean ± SEM.*p < 0.05, **p < 0.01.

Figure S3

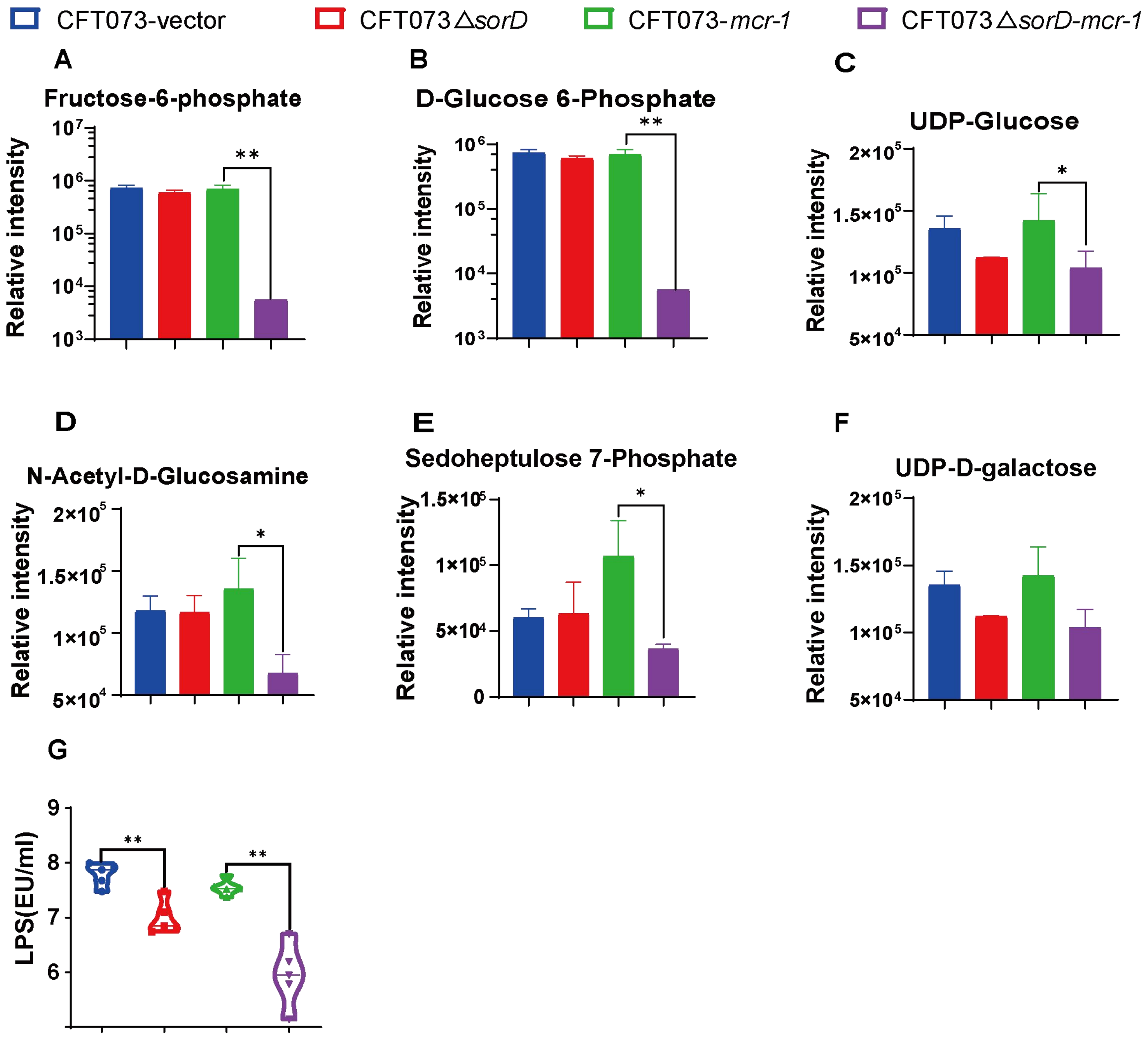

Figure S3. Impaired sorbose metabolism caused defective synthesis of LPS. (A–F) LC-MS analysis of monosaccharide composition of LPS from strains grown in M9 medium supplemented with 2% sorbose (n = 3). (G) The levels of LPS from the strains cultivated in M9 minimal medium with 2% sorbose for 48 h were measured using a chromogenic endotoxin assay (n = 4). All 'n' values represent independent experimental replicates. Data in (A–G) were analysed by Student's *t*-test with Welch's correction or the Mann–Whitney U test for unequal variances. Data are presented as mean ± SEM. $*p < 0.05$, $**p < 0.01$.

Figure S4

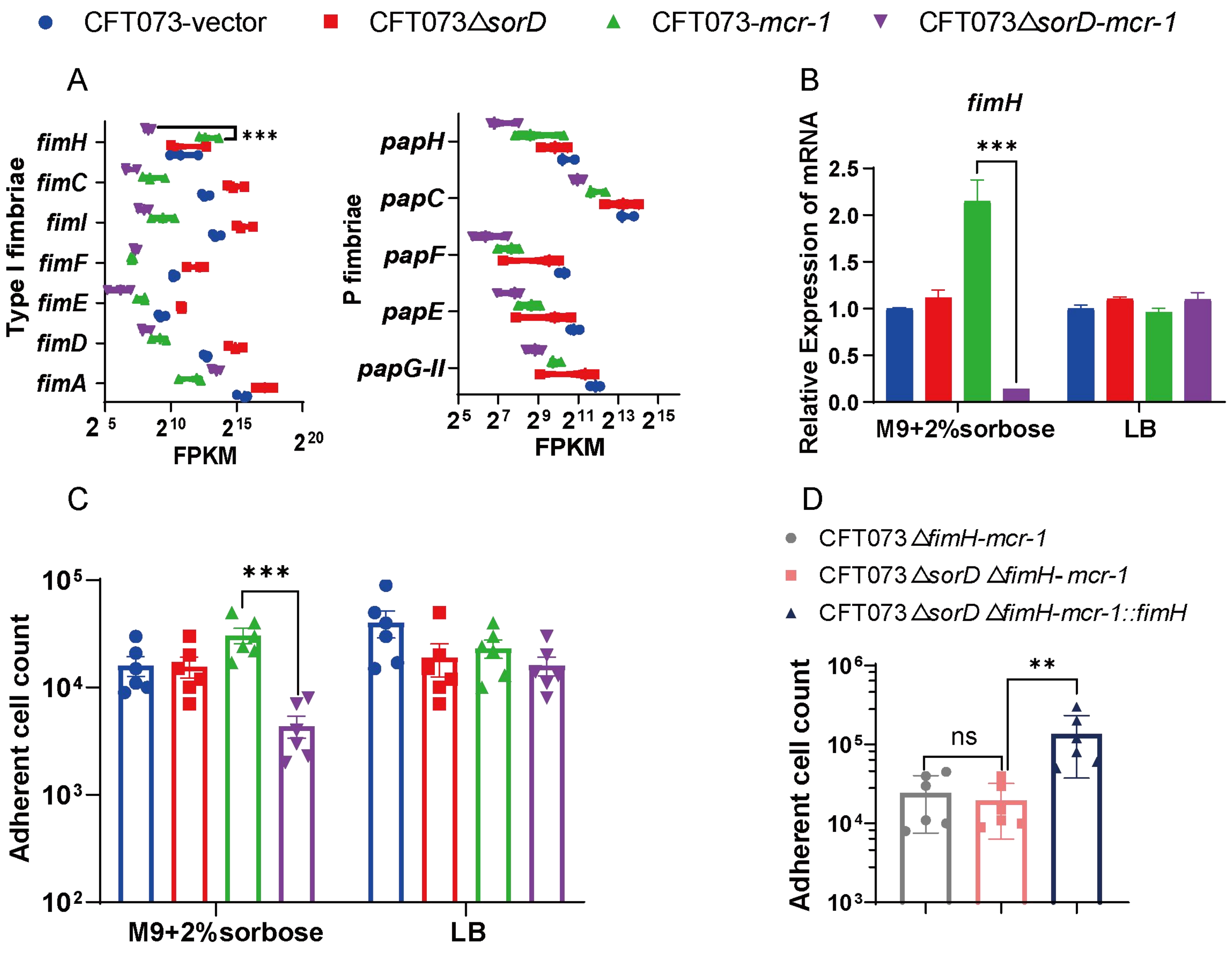

Figure S4. Sorbose metabolism deficiency attenuated MCRPEC virulence through *fimH* downregulation-mediated adhesion impairment. (A) The mRNA levels of type 1 fimbriae (*fim*) and P fimbriae (*pap*) were determined by RNA-seq and are presented as fragments per kilobase of transcript per million mapped reads (FPKM). (B) qPCR validation of *fimH* expression in CFT073 strains (n = 3). (C) Adhesion of strains to SW780 cells after growth in LB or M9 minimal medium with 2% sorbose for 48 h (n = 6). (D) Adherent cell counts of strains to Caco-2 cells (n = 6). All 'n' values represent independent experimental replicates. Data in (A–C) were analysed by Student's *t*-test with Welch's correction for n < 5 or the Mann–Whitney U test for unequal variances. Data in (D) were analyzed by Kruskal-Wallis test followed by Dunn’s test with Bonferroni correction (or one-way ANOVA with Tukey’s HSD when parametric assumptions were met). Data are presented as mean ± SEM. **p < 0.01, ***p < 0.001, ns, not significant.

Figure S5

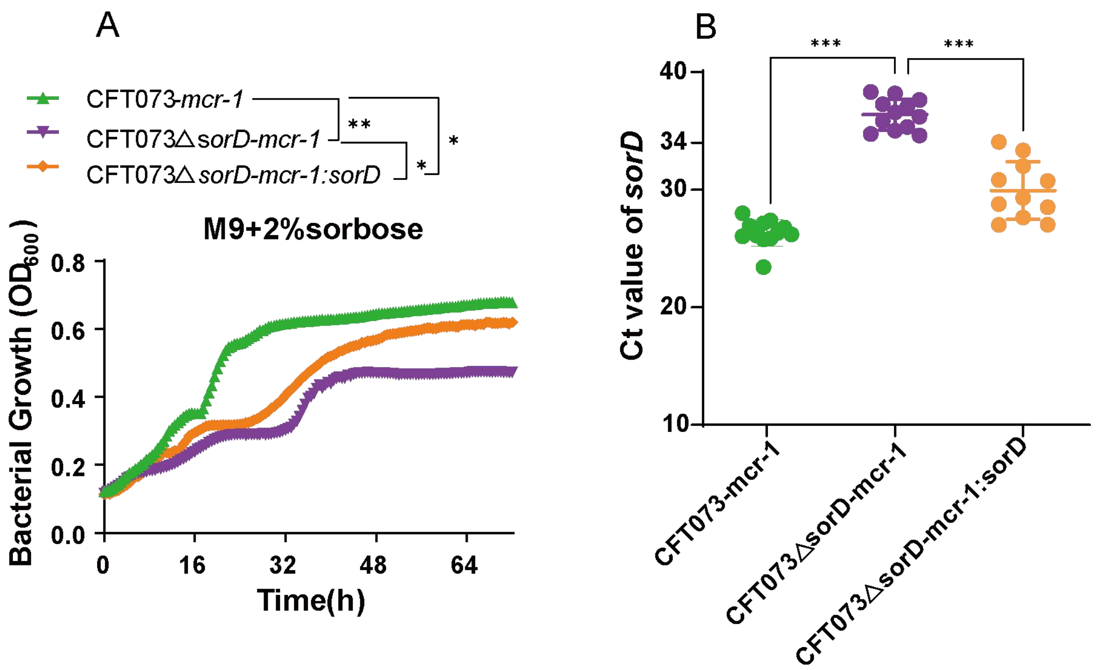

Figure S5. Functional characterization of the *sorD*-complemented strain. (A) Growth kinetics of *E. coli* strains in M9 minimal medium supplemented with 2% sorbose (n = 3). (B) qPCR analysis of *sorD* expression after growth in M9 medium supplemented with 2% sorbose (n = 11–12). All 'n' values represent independent experimental replicates. Data in (A) were analyzed by Kruskal-Wallis test followed by Dunn's test with Bonferroni correction (or one-way ANOVA with Tukey's HSD when parametric assumptions were met). Data in (B) were analysed by Student's *t*-test with Welch's correction or the Mann–Whitney U test for unequal variances. Data are presented as mean ± SEM. $^*p < 0.05$, $^{**}p < 0.01$, $^{***}p < 0.001$.
